# Cellular Target Deconvolution of Small Molecules Using a Selection-Based Genetic Screening Platform

**DOI:** 10.1021/acscentsci.2c00609

**Published:** 2022-09-22

**Authors:** Junxing Zhao, Zhichao Tang, Manikandan Selvaraju, Kristen A. Johnson, Justin T. Douglas, Philip F. Gao, H. Michael Petrassi, Michael Zhuo Wang, Jingxin Wang

**Affiliations:** † Department of Medicinal Chemistry, University of Kansas, Lawrence, Kansas 66047, United States; ‡ Calibr, Scripps Research Institute, La Jolla, California 92037, United States; § Nuclear Magnetic Resonance Laboratory, University of Kansas, Lawrence, Kansas 66047, United States; ∥ Protein Production Group, University of Kansas, Lawrence, Kansas 66047, United States; ⊥ Department of Pharmaceutical Chemistry, University of Kansas, Lawrence, Kansas 66047, United States

## Abstract

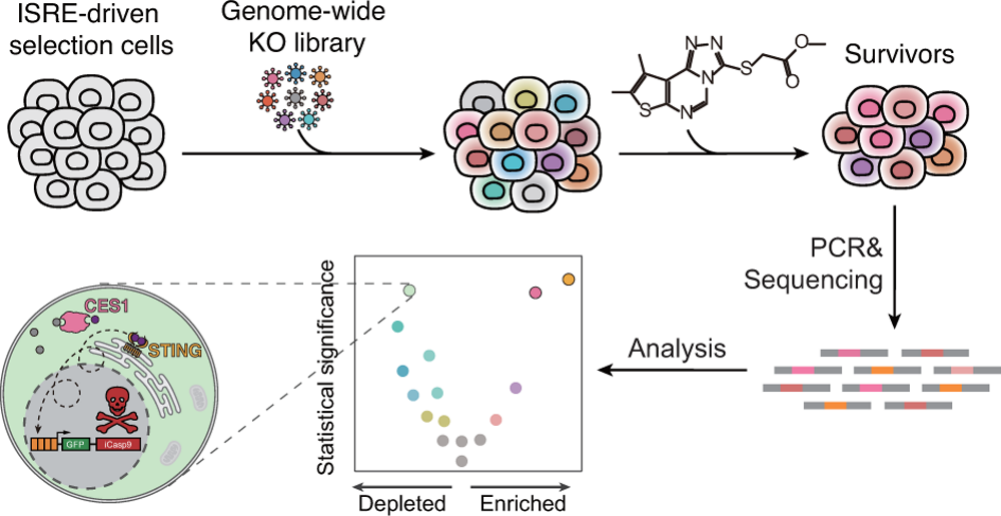

Small-molecule drug target identification is an essential and often rate-limiting step in phenotypic drug discovery and remains a major challenge. Here, we report a novel platform for target identification of activators of signaling pathways by leveraging the power of a clustered regularly interspaced short palindromic repeats (CRISPR) knockout library. This platform links the expression of a suicide gene to the small-molecule-activated signaling pathway to create a selection system. With this system, loss-of-function screening using a CRISPR single-guide (sg) RNA library positively enriches cells in which the target has been knocked out. The identities of the drug targets and other essential genes required for the activity of small molecules of interest are then uncovered by sequencing. We tested this platform on BDW568, a newly discovered type-I interferon signaling activator, and identified stimulator of interferon genes (STING) as its target and carboxylesterase 1 (CES1) to be a key metabolizing enzyme required to activate BDW568 for target engagement. The platform we present here can be a general method applicable for target identification for a wide range of small molecules that activate different signaling pathways.

## Abstract 2

A label-free unbiased drug target identification platform was developed for gene activators by coupling cell survival and genome-wide clustered regularly interspaced short palindromic repeats (CRISPR) loss-of-function screening.

## Introduction

Identification of the cellular targets of bioactive compounds is a crucial step in basic research^1^ and phenotypic drug discovery.^2^ However, the process of target identification is often quite laborious and sometimes fails.^3^ Several strategies for drug target identification have been developed, such as chemical proteomics using affinity probes,^4,5^ thermal proteome profiling,^6−9^ mutagenesis,^10,11^ and genetic screening.^12,13^ One of the most widely adopted methods, chemical proteomics, usually uses chemical probes to pull down the protein targets from the cell lysate, which must subsequently be analyzed and identified by mass spectrometry.^14^ Successful implementation of this approach requires affinity tag-labeled, active chemical probes,^15−18^ which are not always available due to lack of potency or synthetic difficulties, and heavily depends on the abundance of the target protein.^19^ Unlike chemical proteomics, genetic screenings are an unbiased method that disregards the abundance of drug cellular targets and have been extensively used for mechanistic studies of drugs that inhibit cell proliferation or induce cell death.^20−22^ The recent development of clustered regularly interspaced short palindromic repeats (CRISPR)-based approaches has greatly improved the quality of genetic screens in some cases.^23^ Unfortunately, most of these CRISPR-based drug target identifications were limited in utilizing positive selection arising from offsetting the antiproliferative effects of the drugs, such as 6-thioguanine,^24,25^ etoposide,^26^ vemurafenib,^27^ cytosine arabinoside,^28^ and ataxia telangiectasia and Rad3 related (ATR) kinase inhibitors.^29^ To the best of our knowledge, no CRISPR screening strategy has been developed for non-antiproliferative small molecules. We envisioned that a generalizable, CRISPR-based target identification platform for these molecules could be tremendously valuable in the context of the drug development pipelines originating from phenotypic screening campaigns.

**Figure 1:**
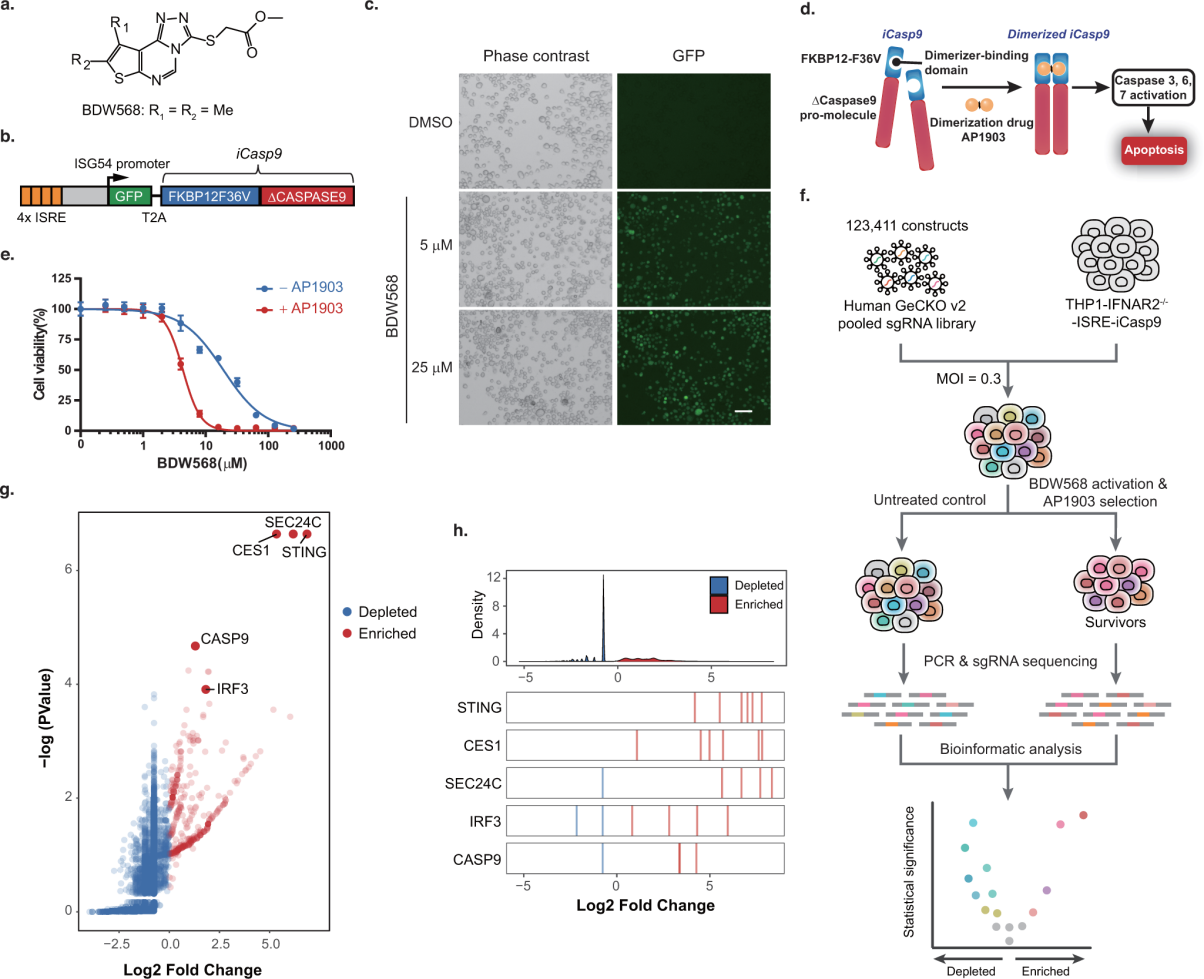
CRISPR-based screening identified STING as the target of BDW568. (a) Chemical structures of BDW568. (b) A suicide gene (iCasp9) controlled by four copies of the ISRE sequence (4× ISRE) with a minimal ISG54 promoter can convert IFN-I signals into iCasp9-triggered cell apoptosis. (c) GFP expression in the selection cells in the presence or absence of BDW568 (16 h). Scale bar = 100 μm. (d) AP1903 dimerizes iCasp9 through the FKBP12^F36 V^ domain, leading to apoptosis of the activated cells. (e) Killing curves of BDW568 in reporter cells in the presence or absence of dimerizer AP1903. Representative curves of two independent experiments; error bars represent ± s.d. for four biological replicates. (f) Schematic diagram illustrates the workflow of genome-wide CRISPR/Cas9 knockout library screening. (g) Scatterplot for genes in BDW568 CRISPR screening (*x*-axis: median of log2 (fold change) for all six sgRNAs per gene; *y*-axis: *P*-value calculated by MAGeCK’s positive algorithm; red dots: enriched genes; blue dots: depleted genes). (h) Top, frequency histogram of sgRNA fold change in the BDW568 group compared to nontreated cells for all sgRNAs. Bottom, distribution of log2 (fold change) for the six sgRNAs targeting candidate genes identified in the BDW568 CRISPR screening (red lines: enriched; blue lines: depleted). Values are averaged over three biological replicates in (g) and (h).

Recently, type-I interferons (IFN-I) agonists that activate the host’s innate and adaptive immune responses have emerged as promising anticancer drug candidates and vaccine adjuvant responses.^30−33^ IFN-I signaling triggers rapid expression of IFN-stimulated genes (ISGs) through activation of the IFN-sensitive response element (ISRE), which in turn regulates the proliferation, differentiation, and functions of immune cells.^34−36^ Using a high-throughput phenotypic IFN-I reporter gene assay, we identified a small molecule, BDW568 (Figure 1a), as a potent IFN-I signaling activator. To identify the specific cellular target of BDW568, a CRISPR-based positive selection platform was tailored to respond to drug-induced IFN-I signaling. Using this platform, we identified and validated STING as the target of BDW568. The CRISPR screen also identified a key metabolizing enzyme CES1 that activates BDW568 in cells. The target identification platform described here is unbiased, modular, and cost-effective, and can be generalizable to all small-molecule drugs that regulate gene expression.

## Results

### Development of CRISPR-Based Target Identification Platform for IFN-I Activators

We identified a new class of IFN-I activators from a luciferase-based high-throughput screening (HTS) in human monocytes (THP-1) that reports for ISRE activation. These compounds feature a tricyclic core with an *S*-acetic ester side chain (Figure 1a) and can robustly induce IFN-I production in ISRE reporter cells and pro-inflammatory cytokines (Supplementary Figure S1). To identify the target of the lead compound, BDW568 (EC_50_ = 5.7 ± 1.3 μM), we established a selection-based CRISPR screen platform for the signaling activation. In this platform, only if the CRISPR-knockout gene impaired the BDW568-induced signaling would the cells survive and proliferate. We envisioned that the identities of the target gene candidates would then be revealed by low-cost sequencing of sgRNA sequences that were integrated into the genomic DNA of the surviving cells.

We first set out to construct a positive selection system, in which BDW568 would activate the expression of the green fluorescent protein (GFP) and a suicide gene named inducible caspase 9 (iCasp9),^37^ through the control of four copies of ISRE and a minimal ISG54 promoter (Figure 1b). This selection system was then lentivirally transduced into THP-1 IFNAR2^–/–^ cells to generate the selection cells. The IFN-I receptor knockout (IFNAR2^–/–^) in THP-1 cells would block complicated IFN-I autocrine and paracrine actions.^38^ A robust positive system expression induced by BDW568 could be visualized by GFP expression in the selection cells. Indeed, BDW568 treatment at 5 and 25 μM resulted in 89% and 94% GFP^+^ cells, respectively, compared to <1% GFP^+^ cells in the DMSO control (Figure 1c). The iCasp9 gene is a fusion of FKBP12^F36 V^ and a truncated human caspase 9 with minimal basal activity (Δcaspase 9), which remains inactive in cells until protein dimerization.^37^ Therefore, the addition of a potent, cell-permeable FKBP12^F36 V^ dimerizer, AP1903 (1 nM), reinforced the dimerization of the cargo Δcaspase 9 in the fusion gene and precisely controlled BDW568-induced apoptosis duration (Figure 1d). In the absence of AP1903, BDW568 is 10-fold less toxic in the selection cells, creating a sufficient window for positive selection (Figure 1e). Together, these results validated the selection cells in both expression and suicide function in the presence of BDW568 and AP1903.

With the validated selection cells in hand, we conducted the CRISPR screening by using a genome-wide human sgRNA library (GeCKO v2) that contains six different constructs of sgRNA for each of the target 19,050 genes.^39^ First, we lentivirally transduced a large number of selection cells (120 × 10^6^) with the sgRNA library at a low multiplicity of infection (MOI = 0.3) to ensure that the cells were transduced with at most one sgRNA sequence per cell and a 50× coverage of the library in the transduced cell pool (Figure 1f). The cells were recovered after transduction in refreshed media and cultured for 8 days before treatment of the compounds. Next, cells were split into selection and nonselection groups in triplicates. The cells in the selection group were exposed to BDW568 (25 μM) for 24 h to fully stimulate IFN-I signaling. The cells were then recuperated for 48 h without BDW568 and subjected to seven cycles of signaling and selection to ensure an enrichment degree of more than 100× before sgRNA quantification by sequencing (Figure 1f; Supplementary Figure S2a). During the selection process, AP1903 (1 nM) was maintained in the cell culture medium (irrespective of the presence or absence of BDW568). The total cell number in the selection group remains nearly constant, whereas the nonselection group showed exponential growth (Supplementary Figure S2b). Finally, the genomic DNAs were individually extracted from the selection and nonselection groups. The vector region in the genomic DNAs containing the sgRNA sequences was amplified by PCR and sequenced (∼500,000 reads per amplicon).

The positive selection enriched the cells with drug target knockout in the pool, and therefore, the target identity could be revealed by comparing the integrated copy number of sgRNA sequences in the selection versus nonselection groups (Figure 1f). For statistical analysis, an algorithm termed model-based analysis of genome-wide CRISPR-Cas9 knockout (MAGeCK)^40^ was used to systematically identify sgRNAs that were enriched or depleted in selection cells relative to nonselection cells (Supplementary Data 1). Using the robust-rank aggregation (RRA) function of MAGeCK on the guide relative enrichments, log2(fold change) was computed to identify genes for which loss-of-function mutations led to enrichment within the selection group over the nonselection group. STING (also known as STING1 or TMEM173) was the top hit in the RRA ranking (Figure 1g, Supplementary Data 1), and six sgRNAs for STING were enriched in BDW568 selection (Figure 1h). STING is a facilitator of innate immune signaling that mediates the response to cytosolic DNAs from bacteria, viruses, and cancer cells, and promotes the production of IFN-I.^41^ Essential genes downstream of STING-ligand binding, such as SEC24C^42^ and IRF3,^43^ were also identified as top-ranked genes in the CRISPR screening (Figure 1g & 1h). SEC24C is required to facilitate STING trafficking from the endoplasmic reticulum (ER) to the Golgi apparatus, which is a key translocation after STING ligand binding.^42^ IRF3 is the transcription factor that controls ISG expression in response to STING signaling.^43^ On the other hand, the genes upstream of STING signaling, such as cyclic GMP-AMP synthase (cGAS),^44^ were not identified. In addition, sgRNAs targeting the suicide gene, caspase 9 (CASP9), are also enriched in the CRISPR screening as expected, indicating that our selection was dependent on the suicide gene function (Figure 1g,h).

### Validation of STING as the Cellular Target of BDW568

**Figure 2:**
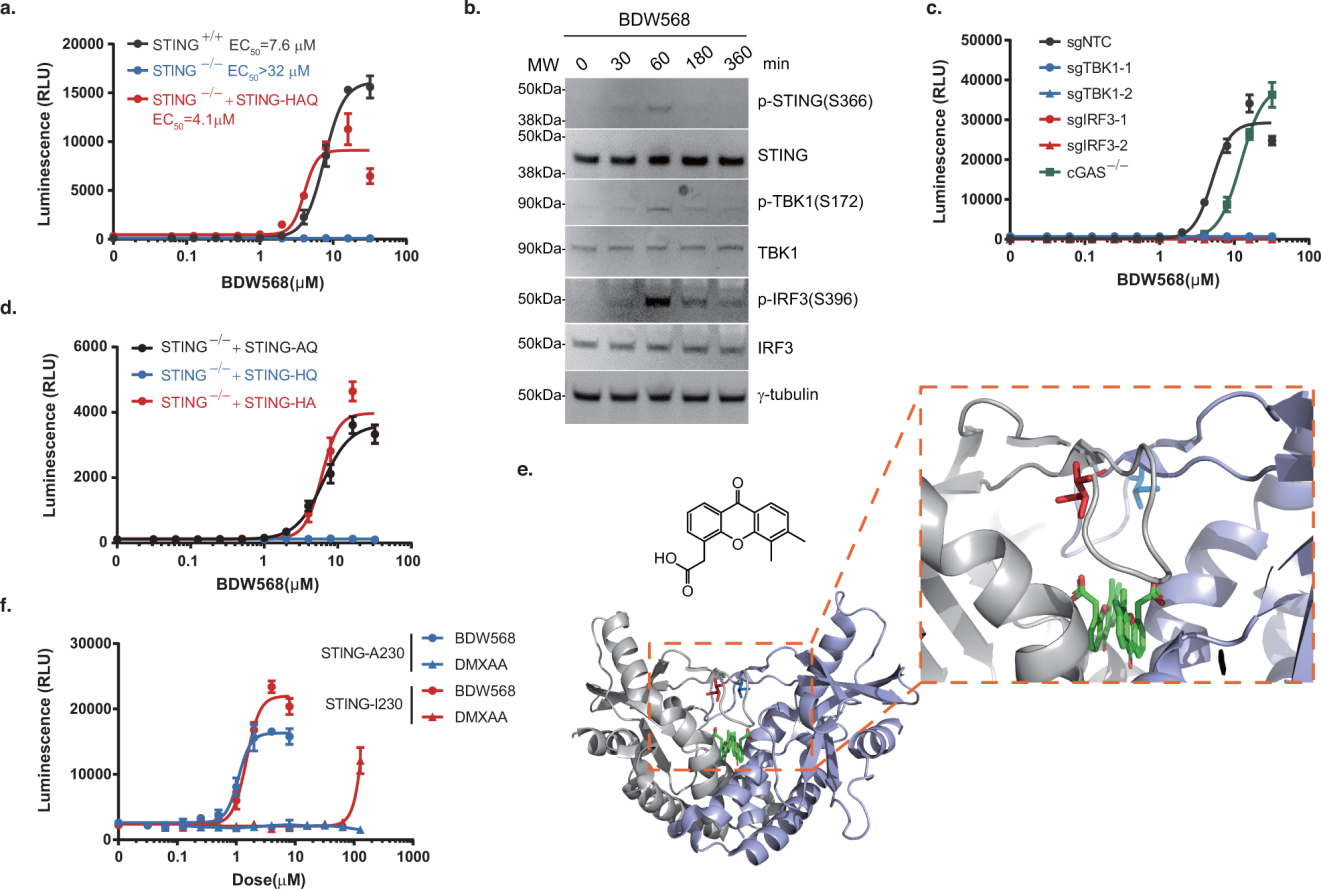
BDW568 activates IFN signaling by targeting STING. (a) Dose–response curves of the BDW568 in THP-1 reporter (STING^+/+^), THP-1 reporter STING knockout (STING^–/–^), and STING-HAQ-expressing cells based on the STING^–/–^ cell line. (b) Immunoblot analysis of the STING pathway in wildtype THP-1 cells treated with 50 μM BDW568 at different time points (loading control, γ-tubulin). Data are representative of two independent experiments. (c) Dose–response curves of BDW568 in TBK1 and IRF3 knockout THP-1 reporter cells using two sgRNA sequences and commercially available THP-1 reporter cGAS^–/–^ cells (NTC, nontargeting control using scrambled sgRNA sequence). (d) Dose–response curves of BDW568 in THP-1 reporter STING^–/–^ cells overexpressing STING-AQ, -HQ, or -HA variants. (e) Chemical structure of DMXAA and crystal structure of DMXAA-bound STING^I230^ (aa 155–341) (PDB: 4QXP). The symmetrical STING^I230^ dimer is shown with individual monomers colored in gray and light blue (residue I230 in each monomer, red and blue sticks; bound DMXAA, green sticks). (f) Dose–response curves of BDW568 and DMXAA in THP-1 reporter STING^–/–^cells overexpressing STING A230 and I230 variants. All dose–response curves are representative of three experiments; error bars represent ± s.d. for four biological replicates.

To validate the role of STING in the BDW568 mechanism of action, we determined the BDW568 activity in the THP-1 STING^–/–^ luciferase reporter cells. As expected, STING knockout almost completely diminished the response to BDW568 (Figure 2a). Overexpression of the THP-1 STING allele (STING with residues H71, A230, and Q293; collectively termed HAQ)^45^ in the STING knockout cells robustly rescued the luciferase signal (Figure 2a). The canonical STING-TBK1-IRF3 phosphorylation axis was also validated by immunoblotting in THP-1 cells with the maximum activation at 60 min drug treatments (Figure 2b). Consistent with this result, knockout of the two essential STING downstream genes, TBK1 and IRF3, also abolished the response to BDW568 in THP-1 cells (Figure 2c). In contrast, the BDW568 activity did not significantly change in the cGAS-knockout reporter cells, indicating that the target of BDW568 is unlikely to be upstream of STING in the pathway. Interestingly, we observed that the residue A230 in the THP-1 STING allele is essential to BDW568 activity. We individually mutated the residues H71, A230, and Q293 in THP-1 STING into R71, G230, and R293 in the STING expression vectors (R71, G230, and R293 residues are more common in the human population)^45^ and tested the BDW568 activity in the STING^–/–^ cells transduced with these vectors. We observed that only STING with A230 (STING-HA and STING-AQ) rescued the reporter signal (Figure 2d). A single G230 mutation in the THP-1 STING allele almost completely abrogated the BDW568 activity (Figure 2d). G230 is located in the interface of the STING dimer and gates the exit tunnel of the bound ligands (Figure 2e).^46^ We hypothesized that a sterically hindered amino acid (i.e., A230) can block the ligand exit and stabilize the ligand binding. This hypothesis is supported by a previous report that the mouse-specific STING agonist, DMXAA,^47^ can also cross-activate human STING having a bulky amino acid residue at position 230 (e.g., G230I single mutation).^46^ As expected, BDW568 was also active on STING with I230 and demonstrated an ∼100-fold higher potency than DMXAA (Figure 2f). Together, we validated that BDW568 is dependent and specific to STING with an A230 residue.

### Carboxylesterase CES1 Is Required to Activate BDW568 for STING Binding

**Figure 3:**
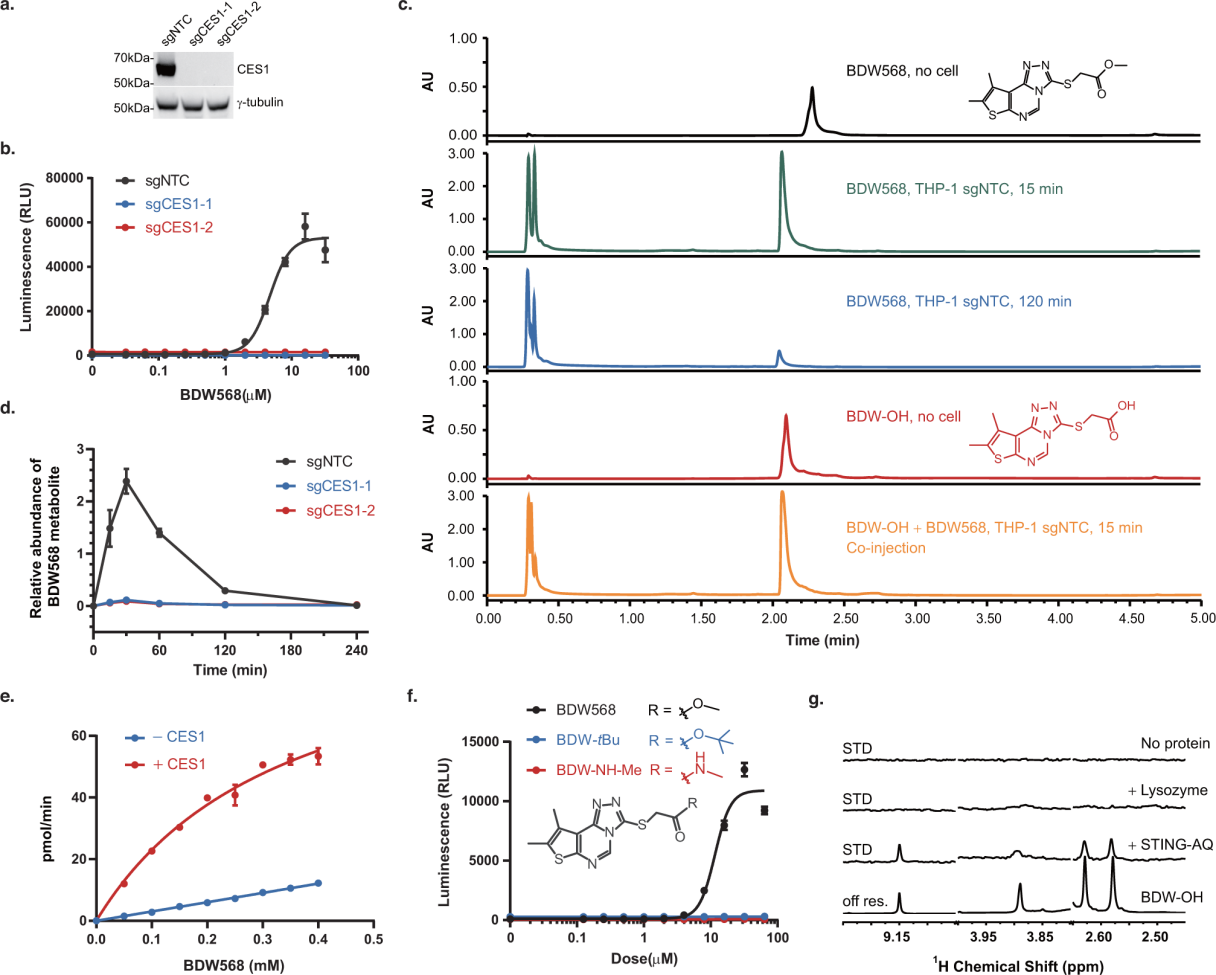
BDW568 as prodrug was metabolized into an active form by CES1. (a) Immunoblotting of single-clone CES1 knockout cells (THP-1 IFNAR2^–/–^) using two sgRNA sequences (loading control, γ-tubulin). Data are representative of two independent experiments. (b) BDW568 activity in single-clone CES1 knockout THP-1 reporter cells using two sgRNA sequences (NTC, nontargeting control using scrambled sgRNA sequence). (c) Ultra-Performance Liquid Chromatography (UPLC) profiles of representative injections of authentic BDW568 and BDW-OH samples (lanes 1 and 4), extracts of BDW568-treated cells at 15 min and 120 min (lanes 2 and 3), and coinjection of authentic BDW-OH and BDW568 metabolite at 15 min (lane 5). (d) BDW-OH-versus-time curve in CES1^+/+^ or CES1^–/–^ THP-1 reporter cells. The error bars represent ± s.d. for biological triplicates of one experiment. (e) Michaelis–Menten curve for recombinant CES1 compared to the enzyme-free reaction in PBS. The hydrolysis of BDW568 was measured in the presence or absence of 100 μg/mL human recombinant CES1 in PBS by LC-MS. Data are presented as mean ± s.d. for biological triplicates from one experiment. (f) Chemical structures and dose–response curves of BDW568, BDW-*t*Bu, and BDW-NH-Me in THP-1 reporter cells. All dose–response curves are representative of three experiments; error bars represent ± s.d. for four biological replicates. (g) STD NMR assay of BDW-OH (300 μM) and STING-AQ (30 μM) in 10% D_2_O, 5% DMSO-*d*_6_ and 10 mM DTT. The assay was performed using a train of low power (50 Hz) Gaussian pulses (total saturation time = 7.5 s) at 30 and 0.85 ppm for off- and on-resonance saturation, respectively. The vertical scale of the STD spectra is increased by a factor of 8 relative to the off-resonance spectrum.

Carboxylesterase 1 (CES1) was identified as a significant hit in the CRISPR screening on BDW568 (Figure 1g,h), which is a serine esterase that is responsible for the hydrolysis of ester-containing xenobiotics.^48^ To validate the role of CES1 in STING activation by BDW568, the top two enriched CES1-targeting sgRNAs from the BDW568 screen were used to stably deplete CES1 in THP-1 reporter cells, and single colonies were subsequently selected and cultured for each sgRNA. The knockout effect was validated by immunoblotting (Figure 3a). As expected, the activity of BDW568 was greatly compromised in both CES1-deficient THP-1 strains (Figure 3b). We envision that CES1 is only required for our STING agonist that contains an ester group. Indeed, other known STING agonists, such as 2′,3′-cGAMP^49^ and SR-717,^50^ which do not have an ester functional group, retained their activity in the two CES1-knockout strains (Supplementary Figure S3). Based on these results, we hypothesized that CES1 is required to hydrolyze the ester group in BDW568 to yield a carboxylic acid for STING activation. In the literature, most STING agonists (e.g., SR-717)^50^ have a carboxylic acid group as an isostere of the phosphate group in the natural STING ligand, 2′,3′-cGAMP. The carboxylic acid group mimics the phosphate and maintains its interactions with STING.^50^ To test the hypothesis, we evaluated the metabolic rate of BDW568 in wildtype THP-1 cells. Interestingly, BDW568 was rapidly metabolized within 15 min, and the retention time of the metabolite is consistent with the authentic hydrolysis product, BDW-OH, in liquid chromatography (Figure 3c, Supplementary Figure S4). BDW-OH was eventually degraded after 2 h in THP-1 cells, suggesting other metabolic pathways exist (Figure 3c). We also tested the BDW568 metabolism in the two CES1 knockout cell lines and did not observe a significant production of BDW-OH (Figure 3d). We have used purified recombinant human CES1 to validate its hydrolytic activity of BDW568. Indeed, although BDW568 can also be slowly hydrolyzed in the enzyme-free PBS buffer, the initial velocity (*v*_0_) is 4.5 times higher in the presence of 100 μg/mL CES1 (see Supporting Information). In other words, CES1 catalyzed the formation of a metabolic more stable compound BDW-OH, which is more resistant to further degradation in cells. Interestingly, BDW-OH per se was not active in THP-1 reporter cells, probably due to poor cellular uptake (Supplementary Table 1). We further replaced the methyl ester group in BDW568 with a nonhydrolyzable *tert*-butyl ester (BDW-*t*Bu) or methyl amide (BDW-NH-Me) and observed no activity in the THP-1 reporter assay (Figure 3e). Together, we concluded that CES1 is an essential metabolizing enzyme required to hydrolyze BDW568 for STING activation.

Next, saturation transfer difference (STD) NMR experiments were carried out to further validate the interaction between BDW-OH and STING protein. The STD assay subtracts a spectrum recorded with selective saturation of protein resonances from the one recorded with off-resonance saturation.^51^ STD NMR is especially useful to illustrate ligand–protein binding with a dissociation binding constant (*K*_d_) within a 10^–3^ to 10^–8^ M range.^51^ We expressed the soluble recombinant STING C-terminal domain (CTD) that contains the ligand binding pocket with A230/Q293 (designate AQ) in *Escherichia coli* (Supplementary Figure S5). Then, the NMR samples were prepared in a 10:1 ligand/receptor ratio. Signals for the aromatic, methylene, and two methyl groups of BDW-OH at 9.15, 3.89, 2.63, and 2.58 ppm, respectively, are present in the off-resonance control spectrum and STD spectrum of samples containing STING-AQ but not observed in control samples prepared using an unrelated protein, lysozyme, or a protein-free control, indicating that this ligand interacts selectively with STING-AQ (Figure 3f).

We also performed the same CRISPR screening with two other validated STING agonists, 2′,3′-cGAMP^49^ and SR-717.^50^ As expected, the screening results also revealed STING as the top target candidate in the presence of 2′,3′-cGAMP or SR-717 selection (Supplementary Figure S2c–f). Other essential genes downstream of STING-ligand binding were also present, such as IRF3 and SEC24C. Pleasantly, because 2′,3′-cGAMP and SR-717 do not require CES1 to activate, no CES1-targeting sgRNAs were enriched in the selected cells treated with these two compounds (Supplementary Figure S2c–f).

## Discussion

In general, transcription activation or suppression is a major category of drug actions to modulate signaling transduction in many cellular physiological and pathological processes.^52^ Our use of a positive selection system for target identification which links transcription activation to a suicide gene is adaptable to a broad range of drugs that activate transcriptional responses. This is a significant extension of the existing reported CRISPR screening systems that were previously only limited to antiproliferative drugs.^24−29^ We envision that the suicide gene can also be further replaced by an antibiotic resistant gene to generalize the selection system for drugs that suppress transcriptional responses. We have applied this selection-based CRISPR screening platform and successfully determined STING to be the specific cellular target of a phenotypic HTS lead, BDW568, a potent activator of IFN-I signaling. Compared to the traditional chemical proteomic method, the selection platform does not require any modification on the small molecule (i.e., label-free). This feature is particularly advantageous for BDW568, on which most chemical modifications abolished the IFN-I activation activity (Supplementary Table 1). Due to the lack of active chemical probes in the BDW568 scaffold, the chemical proteomic approach is virtually not applicable for target identification. On the other hand, the selection-based CRISPR screening also revealed essential genes downstream of the drug-cellular target binding event (e.g., SEC24C, IRF3 in this report). These downstream “hits” will also serve as evidence for pharmacological engagement of a specific signaling pathway. In practice, the identification of a cohort of genes in the same signaling pathway from the CRISPR screening will greatly narrow down the list of candidate targets and reduce the validation effort. As demonstrated in this report, the direct target of the compound is likely to be the most upstream protein of the identified genes in the engaged signaling pathway (e.g., STING is upstream to SEC24C and IRF3). We also identified an essential metabolizing enzyme that activates BDW568 (i.e., CES1) in this work, indicating a wide utility of the CRISPR screening in pharmacological investigations.

Recently, STING agonists have attracted much attention due to their synergy with other anticancer immunotherapies arising from enhanced type-I interferon signaling. For example, the combination of programmed death (PD)-1 blocker and a STING agonist almost fully inhibited the solid tumor that was insensitive to single-agent treatment in a mouse model.^53^ In the past few years, several synthetic compounds with more advantageous pharmacokinetic properties were also developed.^50,54−56^ To our knowledge, BDW568 is the first STING agonist that critically depends on residue A230 in STING. In the future, chemical modifications will be performed to BDW568 to improve its target selectivity and metabolic stability.

In summary, we have presented a label-free, unbiased drug target identification platform that uses a pathway-specific selection system coupled with CRISPR-based loss-of-function screening. We envision that this selection-based target identification platform will be widely useful in target identification for drugs that regulate gene expression and bring about a paradigm shift in mechanistic studies in phenotypic drug discovery.

## Methods

### Cell Lines, Cell Culture, and CRISPR sgRNA Library

Wildtype THP-1 cells (TIB-202) were obtained from ATCC. THP-1 ISRE reporter cells, including THP-1-Dual (thpd-nfis), THP-1-Dual KO-STING (thpd-kostg) THP-1-Dual KO-cGAS (thpd-kocgas), and THP-1-Dual KO-IFNAR2 (thpd-koifnar2) cells, were obtained from InvivoGen. Wildtype and reporter THP-1 cells were grown at 37 °C in the full medium containing RPMI 1640 (Gibco, 31-800-022), 10% fetal bovine serum (HyClone, SH3039603HI), 2 g/L sodium bicarbonate, 1 mM sodium pyruvate, 10 mM HEPES buffer (pH 7.3), 1× Antibiotic-Antimycotic (Gibco, 15-240-062), and 1 mM β-mercaptoethanol. Human GeCKOv2 CRISPR knockout pooled library was a gift from Feng Zhang (Addgene, #1000000048).

### THP-1 ISRE Reporter Assay

THP-1 ISRE reporter cells were seeded in 384-well plates with 5000 cells per well in a total of 30 μL of full medium. Compounds were incubated with the reporter cells for 48 h before 10 μL of 0.5 × QUANTI-Luc (InvivoGen, rep-qlc2) was added to each well. The plates were measured immediately using a luminescence plate reader. To determine dose responses, the cells were treated with a serial dilution of BDW568 (or analogues) at 12 concentrations in quadruplicate.

### Preparation of the Selection Cells

To construct the selection system, the ISG54 promoter^57^ and iCasp9 sequences were synthesized and inserted into the pGreenFire1-ISRE-Neo vector (System Biosciences, TR016PA-N) and replaced the original sequences of mCMV promoter and luciferase, respectively. Then the whole selection cassette (4 × ISRE-ISG54-dscGFP-T2A-iCasp9) was cloned and inserted into the lentiviral vector pCDH-CMV-MSC-EF1α-Hygro (System Biosciences, CD515B-1) to replace the original CMV promoter. To pack the selection system in VSVG-pseudotyped lentiviral particles, the selection plasmid and packaging plasmids pMD2.G, psPAX2 were cotransfected with polyethylenimine (Polysciences, linear, MW ≈ 25000) in 293FT cells (Thermo Scientific, R70007). After 48 h, the virus-containing supernatant was collected and spun for 10 min at 4 °C (500*g*) to remove cells. Lentiviral particles were concentrated ∼10× in volume with a Lenti-X Concentrator reagent (Clontech, PT4421-2). THP-1-Dual KO-IFNAR2 cells were transduced by addition of concentrated viral suspension at a multiplicity of infection (MOI) of 1 in the full medium containing 8 μg/mL Polybrene (Sigma-Aldrich, TR-1003-G). After a 24 h of incubation at 37 °C, the virus-containing medium was removed and replenished with a fresh medium. After an extra 24-h recovery, the transduced cells were selected in 1 μg/mL hygromycin (Cayman, 14291) for 7 days.

### Genome-wide CRISPR Screen

CRISPR sgRNA library amplification, lentiviral package, titration, and transduction were performed in triplicates according to a standard protocol.^58^ Briefly, 120 million THP-1 selection cells (20 million cells per sample, ∼50× coverage of library) were infected with the lentiviral sgRNA library at a multiplicity of infection (MOI) of 0.3. Infected cells were selected with puromycin (0.6 μg/mL) at 48 h postinfection. On day 7, puromycin containing medium was replaced with fresh medium for a 24-h recovery. Drug selection was performed by adding BDW568 (or other IFN-I activators) with seven cycles of the following treatment: 25 μM BDW568 treatment for 1 day followed by a 2-day recovery without the compound; 30 μM 2′,3′-cGAMP treatment for 2 days followed by a 1-day recovery without the compound; 10 μM SR-717 treatment for 1 day followed by a 2-day recovery. AP1903 (1 nM) was present in the medium throughout the selection process. The control group was cultured without any drug treatment. After selection, apoptotic cells were removed by using a dead cell removal kit (Miltenyi Biotec, 130-090-101) in the BDW568 and other IFN-I activator groups. Genomic DNA was isolated from live-cell pellets using DNeasy Blood & Tissue kits (Qiagen, 69504). PCR amplification of sgRNAs was performed using AccuPrime pfx DNA polymerase (Thermo Scientific, 12344032) and vector-specific primers (Forward: 5′-AATTTCTTGGGTAGTTTGCAGTT, Reverse: 5′-GACTCGGTGCCACTTTTTCAA). Eight PCRs in 50 μL reactions were performed for each sample with 1 μg of genomic DNA template per reaction with the following cycling conditions: 15 s at 95 °C, 30 s at 62 °C, and 30 s at 68 °C, 24 cycles. The PCR product in each sample was pooled and purified by NucleoSpin Gel and PCR Clean-up kit (Takara Bio, 740611.250) according to the manufacturer’s protocol. The DNA amplicon was submitted for next-generation sequencing (GENEWIZ, Amplicon-EZ, $80 per sample for academic users).

### DNA Library Preparation and Illumina Sequencing

DNA library preparations, sequencing reactions, and adapter sequences trimming were conducted at GENEWIZ, Inc. (South Plainfield, NJ, USA). DNA Library Preparation were performed using NEBNext Ultra DNA Library Prep kit following the manufacturer’s recommendations (Illumina, San Diego, CA, USA). Briefly, end repaired adapters were ligated after adenylation of the 3′-ends followed by enrichment by limited cycle PCR. DNA libraries were validated and quantified before loading. The pooled DNA libraries were loaded on the Illumina instrument according to manufacturer’s instructions. The samples were sequenced using a 2× 250 paired-end (PE) configuration. Image analysis and base calling were conducted by the Illumina Control Software on the Illumina instrument. The raw Illumina reads were checked for adapters and quality via FastQC. The raw Illumina sequence reads were trimmed of their adapters using Trimmomatic v. 0.36. Raw sequence data (.bcl files) generated from Illumina MiSeq were converted into fastq files and demultiplexed using Illumina bsl2fastq v. 2.17 program. Raw sequence data were demultiplexed using bcl2fastq version 2.17.1.14. Read pairs were trimmed for adapter sequences and low-quality base calls using Trimmomatic version 0.36. Reads were discarded if they were less than 30 bases long.

### IFN-I Stimulated Gene Expression

Wildtype THP-1 cells were treated with 50 μM BDW568, and the cells were harvested at different time points as indicated (Supplementary Figure S1). The total RNA was extracted using the RNeasy mini kit (Qiagen, 74106) using the manufacturer’s protocol. 0.5 μg of total RNA was reverse-transcribed to cDNA by M-MLV reverse transcriptase (Promega, M1701). Gene expression was assessed using primers listed below with a 2× SYBR Green master mix (APExBIO, K1070). Gene expression was normalized with the endogenous GAPDH level and was reported as fold changes in mRNA expression. See Supplementary Data 2 for primer sequences.

### Drug-Induced Killing Curves

THP-1-Dual IFNAR2KO reporter cells were seeded in a 384-well plate with 5000 cells/well in 30 μL of medium with or without AP-1903. They were treated with a 1:2 serial dilution of BDW568 at 12 concentrations in quadruplicates. Cell viability was assessed after 48 h incubation with CellTiter-Glo (G9242, Promega). Relative viability was normalized to DMSO treated control and determined CC_50_ by nonlinear regression in GraphPad Prism 7.

### Chemical Synthesis

See Supplementary Data 3 for synthetic protocols, characterizations, and NMR spectra.

### Plasmid Construction

(a) Plasmids for gene knockout. Individual sgRNA sequences were cloned by ligating annealed oligonucleotides into a lentiCRISPRv2 vector (Addgene, 52961) according to the literature protocol.^39^ See Supplementary Data 2 for sgRNA sequences used in the manuscript. (b) Plasmids for STING variants. The STING/STING-HAQ sequence was cloned from pTRIP-SFFV-mtagBFP-2A-hSTING (Addgene, 102586) or pTRIP-SFFV-mtagBFP-2A-STINGHAQ (Addgene, 102587) and inserted into the lentiviral vector pLVX-IRES-Puro (Clonetech, 632183) between *Eco*RI and XbaI using an In-Fusion cloning kit (Takara Bio, 639649). STING variants were constructed by PCR-based site-directed mutagenesis (see Supplementary Data 2 for primer sequences). The mutated STING expression vectors were treated with DpnI (FD1704, Thermo Scientific) to remove plasmid templates and then transform into component cells, followed by Sanger sequencing validation. (c) Plasmids for recombinant STING-AQ CTD production in *E. coli*. The gene sequence encoding the C-terminal domain (residues 139–379) of human STING-AQ was synthesized by gBlock at Integrated DNA Technologies and inserted into the pET-28a vector in the restriction site *Eco*RI.

### Recombinant Protein Production and Purification

Recombinant 6× His tagged STING-AQ was expressed and purified as summarized. Plasmids for STING-CTD and STING-AQ expression were transformed into BL21 (DE3) pRARE chemically competent cells. Overnight cultures of the resulting transformed bacteria were inoculated into 1 L of LB broth at a 1:20 dilution. Bacterial cultures were grown to OD600 0.6–0.8 at 37 °C, after which protein expression was induced by the addition of 0.4 mM isopropyl β-d-1-thiogalactopyranoside (IPTG) for 12 h at 30 °C. Bacteria were harvested by centrifugation and went through three repeats of freeze–thaw cycles in −80 °C and a water bath at room temperature. The bacterial pellets were then resuspended in Buffer A (50 mM Tris-HCl pH8.0, 500 mM NaCl). Cells were lysed by sonication, and clarified lysate was loaded onto a HisTrap HP column (GE Healthcare, 17524801) installed on an AKTA Pure Purification program. The protein was eluted with a gradient of Buffer A and Buffer B (Buffer A with 500 mM imidazole). The resulting purified proteins were analyzed by SDS-PAGE Coomassie staining (Supplementary Figure S5) and exchanged into PBS buffer by dialysis.

### Saturation Transfer Difference NMR Assay

NMR tubes were purchased from Wilmad-Lab Glass (Vineland, NJ). Solvents were purchased from Cambridge Isotopes (Tewksbury, MA). NMR sample concentrations were equal to 300 and 30 μM for the ligand and protein, respectively, with 10% D_2_O, 5% DMSO-*d*_6_, and 10 mM DTT (added immediately prior to data collection) in PBS. NMR data were acquired using a Bruker AVIII 600 MHz spectrometer equipped with a 5 mm TXI probe. Specific acquisition parameters are as follows: Bruker pulse program ‘stddiffesgp.2′ with minor modifications to facilitate automated acquisition; acquisition time per scan, 2.10 s; spectral width, 13.0 ppm; delay between scans, 15 s; total number of scans, 256 at each saturation frequency; total experiment time, 2.5 h; 1H excitation power, 23.8 kHz; spin lock power, 9.6 kHz; saturation pulse, a train of 50 ms Gaussian pulses; saturation time, 7.5 s; saturation power, 50 kHz; saturation frequency, 30 and 0.85 ppm. Data were processed using the Bruker automation script “stdsplit” with minor modifications to facilitate automated processing and visualized using MestreNova software.

### Measurement of Drug Metabolism

CES1 knockout cells as well as their parental THP-1-IFNAR2KO cells (10^6^) were treated with 50 μM BDW568 for the indicated time. Cells were collected and washed by 1× PBS once, and then 50% acetonitrile was added and vortexed for 1 min. Samples were centrifuged at 17,000 *g* for 10 min to remove insoluble cell debris. Compound **4** (0.5 μL at 1 mM; see Supplementary Information) was added to the supernatant of each sample (19.5 μL) as an internal standard for UPLC-MS analysis. Ten microliters of the mixed solution were injected into UPLC-MS for analysis with the following conditions: instrument: Waters ACQUITY UPLC H Class Plus in tandem with a Qda mass detector; column: ACQUITY UPLC BEH C18 1.7 μm (21 × 50 mm); mobile phase: acetonitrile and 0.1% formic acid water solution; gradient: 0 min, 2% acetonitrile; 3 min, 98% acetonitrile; 5 min, 2% acetonitrile; UPLC-MS results were analyzed using Waters Empower 3 software.

### In Vitro Metabolite Study of BDW568 with Recombinant CES1

Recombinant human CES1 protein expressed in *E. coli* was purchased from Creative BioMart (CES1-106H) as a 10 mg/mL stock solution. Before use, CES-1 protein was thawed on ice and diluted in the working buffer (1× PBS (pH 7.4)/acetonitrile, 98/2 (v/v)). To make a 100 μg/mL working solution, 10 μL of CES1 stock solution and 10 μL of ATP (100 mM) were diluted in the working buffer (1 mL). A solution without CES1 protein was also prepared as a blank control. BDW568 were prepared as 8 mM, 7 mM, 6 mM, 5 mM, 4 mM, 3 mM, 2 mM, and 1 mM solution in DMSO. One microliter of each BDW568 solution was added to 19 μL working solution and incubated at 37 °C for 1 h. Twenty microliters of ethanol were then added to stop the reaction by denaturing CES1 protein. The supernatant (10 μL) was used for UPLC-MS analysis. The ratio between metabolite and BDW568 was determined and used to calculate the reaction rate (pmol/min). The Michaelis–Menten curves were plotted using GraphPad Prism 8. UPLC-MS conditions: instrument: Waters ACQUITY UPLC H Class Plus in tandem with a Qda mass detector; column: ACQUITY UPLC BEH C18 1.7 μm (21 × 50 mm); mobile phase: Acetonitrile (organic phase) and 0.1% formic acid water solution; gradient: 0 min, 2% acetonitrile; 3 min, 98% acetonitrile; 5 min, 2% acetonitrile. UPLC-MS results were analyzed using Waters Empower 3 software.

### Western Blotting

Two million cells of each sample were lysed in 50 μL of 1× RIPA buffer (Cell Signaling Technology, 9806) with freshly added 1 mM PMSF and 1× Halt phosphatase inhibitor cocktail (Thermo Scientific, 78420). The cell lysate was vortexed thoroughly for 30 s and centrifuged at 17000*g* at 4 °C for 10 min to remove cell debris. The supernatant was mixed with 2× SDS sample buffer (100 mM Tris-HCl (pH 6.8), 4% SDS, 0.2% bromophenol blue, 20% glycerol, 200 mM β-mercaptoethanol) and heated at 95 °C for 10 min. Western blotting was performed using Bolt 4–12% Bis-Tris gels and an iBolt 2 transfer system following the manufacturer’s instructions (Thermo Scientific). Primary antibody was incubated overnight at 4 °C with shaking. Primary antibody information and dilution factor are listed in Supplementary Data 2. Fluorescent secondary antibodies, goat anti-rabbit IgG Alexa Flour Plus 800 (Thermo Scientific, A32735), and goat anti-mouse IgG Alexa Flour Plus 680 (Thermo Scientific, A32739) were diluted in a 1:2500 ratio and incubated for 2 h at room temperature with shaking.

### Safety Statement

No unexpected or unusually high safety hazards were encountered in this study.

## Notes

All data needed to evaluate the conclusions in the paper are present in the paper and/or the Supporting Information. The sequencing raw data are deposited in the Sequence Read Archive (SRA) of National Center for Biotechnology Information (NCBI) (Accession # PRJNA836618).

## Supporting Information Available

Supplementary Data 1: CRISPR screening results (XLSX)
Supplementary Data 2: Primers and sequences (PDF)
Supplementary Data 3: Chemicals, Syntheses, and Characterizations (PDF)
Supplementary Figures S1–5, Supplementary Tables S1–2 (PDF)
