# Supplementary material for: Cellular Target Deconvolution of Small Molecules Using a Selection-Based Genetic Screening Platform
Source: ACS Cent Sci. 2022 Sep 22;8(10):1424–34. doi: 10.1021/acscentsci.2c00609 (PMC9615120; doi:10.1021/acscentsci.2c00609)
Supplement: Supplementary file 2 — oc2c00609_si_002.pdf [file oc2c00609_si_002.pdf]

Primers for RT-qPCR:

CXCL10_F	5'-AGTGGCATTCAAGGAGTACC
CXCL10_R	5'-TGATGGCCTTCGATTCTGGA
IFNA1_F	5'-GCCTCGCCCTTTGCTTTACT
IFNA1_R	5'-CTGTGGGTCTCAGGGAGATCA
ISG54_F	5'-AACCTACTGGCCTATCTAAAGC
ISG54_R	5'-CATGCTCTTGCTGGATTAACTC
IL6_F	5'-CACTGGTCTTTTGGAGTTTGAG
IL6_R	5'-GGACTTTTGTACTCATCTGCAC
GAPDH_F	5'-GAGAAGGCTGGGGCTCATTT
GAPDH_R	5'-CAGGAGGCATTGCTGATGAT

The sgRNA sequences:

NTC	HGLibA_64384	5'-ACGGAGGCTAAGCGTCGCAA
CES1-1	HGLibB_09209	5'-TCCTCCTAAGTATTTCTCAG
CES1-2	HGLibA_09217	5'-GTGCGGCATCAACCTATGAT

Primers for site-specific mutagenesis:

H71R_F	5'-TGAGGAGCTGCACCACATCCACTCCAGGTACCGGGGCA
H71R_R	5'-TGGATGTGGTGCAGCTCCTCAGCCAGGCTGCAGACCCCGTT
A230G_F	5'-AGCAGACCGCTGACCGTGCTGGCATCAAGGATCGGGTTTA
A230G_R	5'-GCACGGTCAGCGGTCTGCTGGGGCAGTTTATCCAGGAA
Q293R_F	5'-TCTTCTGCCAGACACTTGAGGACATCCTGGCAGATGC
Q293R_R	5'-TCAAGTGTCTGGCAGAAGAGTTTGGCCTGCTCAAGCCTATC
G230A_F	5'-CAGACCGCTGACCGTGCTGGCATCAAGGATCGGGTTTA
G230A_R	5'-CACGGTCAGCGGTCTGCTGGGGCAGTTTATCCAGGAA
G230I_F	5'-CAGACCATTGACCGTGCTGGCATCAAGGATCGGGTTTA
G230I_R	5'-CACGGTCAATGGTCTGCTGGGGCAGTTTATCCAGGAA

gBlock sequence for STING-AQ:

ATGCTGGCCCCAGCTGAGATCTCTGCAGTGTGTGAAAAAGGGAATTTCAACGTGGCCCATGGGCTG
GCATGGTCATATTACATCGGATATCTGCGGCTGATCCTGCCAGAGCTCCAGGCCCGGATTCGAACTT
ACAATCAGCATTACAACAACCTGCTACGGGGTGCAGTGAGCCAGCGGCTGTATATTCTCCTCCCATT
GGACTGTGGGGTGCCTGATAACCTGAGTATGGCTGACCCCAACATTCGCTTCCTGGATAAACTGCCC
CAGCAGACCGCTGACCATGCTGGCATCAAGGATCGGGTTTACAGCAACAGCATCTATGAGCTTCTG
GAGAACGGGCAGCGGGCGGCACCTGTGTCCTGGAGTACGCCACCCCCTTGCAGACTTTGTTTGCCA
TGTCACAATACAGTCAAGCTGGCTTTAGCCGGGAGGATAGGCTTGAGCAGGCCAAACTCTTCTGCCA
GACACTTGAGGACATCCTGGCAGATGCCCCTGAGTCTCAGAACAACTGCCGCCTCATTGCCTACCAG
GAACCTGCAGATGACAGCAGCTTCTCGCTGTCCCAGGAGGTTCTCCGGCACCTGCGGCAGGAGGAA
AAGGAAGAGGTTACTGTGGGCAGCTTGAAGACCTCAGCGGTGCCCAGTACCTCCACGATGTCCCAA
GAGCCTGAGCTCCTCATCAGTGGAATGGAAAAGCCCCTCCCTCTCCGCACGGATTTCTCTTGA

Primary antibody order information and dilution factor in immunoblotting:

Antibody	Vendor	Catalog #	Dilution factor
STING	Cell Signaling Technology	13647	1:1000
p-STING(S366)	Cell Signaling Technology	85735	1:500
IRF3	Cell Signaling Technology	4302	1:750
p-IRF3(S396)	Cell Signaling Technology	29047	1:500
TBK1	Cell Signaling Technology	3504	1:750
p-TBK1(S172)	Cell Signaling Technology	5483	1:500
γ-tubulin	Sigma-Aldrich	T6557	1:5000
CES1	Proteintech	16912-1-AP	1:1000
