# Supplementary material for: Cellular Target Deconvolution of Small Molecules Using a Selection-Based Genetic Screening Platform
Source: ACS Cent Sci. 2022 Sep 22;8(10):1424–34. doi: 10.1021/acscentsci.2c00609 (PMC9615120; doi:10.1021/acscentsci.2c00609)
Supplement: Supplementary file 3 — oc2c00609_si_003.pdf [file oc2c00609_si_003.pdf]

Chemicals, Syntheses and Characterizations

Reagents and solvents were purchased from commercial sources (Fisher, Sigma-Aldrich and Combi-Blocks) and used as received. Reactions were tracked by TLC (Silica gel 60 F_{254}, Merck) and Waters ACQUITY UPLC H Class Plus with QDA Mass Detector. Intermediates and products were purified by a Teledyne ISCO Combi-Flash system using pre-packed silica gel cartridges. NMR spectra were acquired on a Bruker AV400 instrument (400 MHz for ^{1}H NMR, 100 MHz for ^{13}C NMR) or Bruker AV500 instrument (500 MHz for ^{1}H NMR, 126 MHz for ^{13}C NMR). NMR data were recorded as follows: chemical shift (δ) in ppm, coupling constant (J) in Hz, multiplicity (s = singlet, d = doublet, t = triplet, sep = septet, m = multiplet or overlap of nonequivalent resonances). The ^{13}C NMR spectra were obtained with H decoupling. MS-ESI spectra were recorded on Waters QDA Mass Detector.

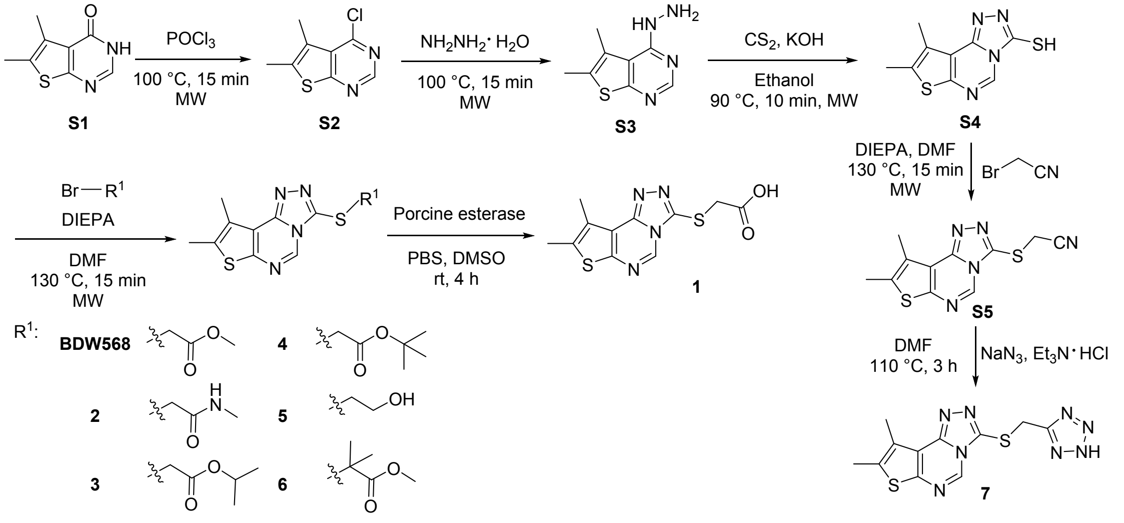

Scheme 1: Chemical modifications on the side chain of **BDW568**.

A. Syntheses of intermediate S4[1]

Step 1: Compound **S1** (100 mg, 0.56 mmol) in $POCl_3$ (0.5 mL) was heated at 100 °C for 15 min under microwave irradiation. $POCl_3$ was evaporated in vacuo and the residual material was re-dissolved in ethyl acetate. After washing with saturated $NaHCO_3$ solution, the organic phase was collected and dried with Na_2SO_4 and concentrated in vacuo to furnish compound **S2**. The crude compound was purified by silica gel column chromatography using 0–10% ethyl acetate in hexanes. Off-white solid, 0.10 g (yield: 92 %).

Step 2: Compound **S2** (100 mg, 0.5 mmol) in hydrazine hydrate (1 mL) was heated at 100 °C for 15 min under microwave irradiation. The solid formed was filtered and washed with water to remove excess hydrazine. The water residue was removed by co-evaporating with toluene. The crude material was used in the next step without further purification. Yellow solid, 60 mg (yield: 59%).

Step 3: A solution of KOH (22.4 mg, 0.4 mmol) and CS_2 (114 mg, 1.5 mmol) in ethanol was added dropwise to the solution of compound **S3** (60 mg, 0.3 mmol) in ethanol. The reaction mixture was then heated to 90 °C for 10 min under microwave irradiation. The solvent was removed in vacuo and the residual material was re-dissolved in water and acidified with 1N HCl. The precipitation formed was filtered and co-evaporated with toluene to remove water residue. To further purify the compound, the crude solid was heated and recrystallized in ethanol. Yellow solid, 55 mg (yield: 76%). ^{1}H NMR (500 MHz, $DMSO_{d6}$) δ 14.70 (s, 1H), 8.87 (s, 1H), 2.49 (s, 3H), 2.46 (s, 3H); ^{13}C NMR (126 MHz, $DMSO_{d6}$) δ 159.9, 149.8, 142.9, 135.6, 135.2, 127.1, 118.0, 13.1, 12.6. Mass ESI $[M+H]^+$ = 237.07.

B. Syntheses of BDW568, BDW-OH (1), BDW-NHMe (2), compound 3, BDW-OBu (4), and compounds 5–7

Compound **S4** (20.0 mg, 1.0 equiv.) in DMF was added the respective alkyl bromide (1.5 equiv.) followed by *N*, *N*-diisopropylethylamine (DIPEA, 1.5 equiv.) and the reaction mixture was heated at 130 °C for 15 min under microwave irradiation. The reaction mixture was washed by water and extracted with ethyl acetate (2 × 20 mL). The combined organic layer was washed with water followed by brine and was dried with Na_2SO_4. The solution was filtered and concentrated, and the crude product was purified by silica-gel column chromatography with 0–40% ethyl acetate in hexanes.

Methyl 2-((8,9-dimethylthieno[3,2-e][1,2,4]triazolo[4,3-c]pyrimidin-3-yl)thio)acetate (BDW568)
Light yellow solid, 13.6 mg (52%). ^{1}H NMR (500 MHz, $CDCl_3$) δ 8.88 (s, 1H), 4.02 (s, 2H), 3.69 (s, 3H), 2.72 (s, 3H), 2.55 (s, 3H); ^{13}C NMR (126 MHz, $CDCl_3$) δ 168.9, 149.6, 148.3, 139.9, 136.8, 132.2, 128.1, 120.0, 53.2, 36.7, 13.8, 13.5. Mass ESI $[M+H]^+$ = 308.98.

To synthesize **BDW-OH**, a solution of **BDW568** (10.0 mg, 0.03 mmol) in DMSO (0.15 mL, 5% in total volume) was added 1× PBS buffer (pH 7.4, 2.85 mL) followed by esterase from porcine liver (21 mg, 7 mg per mL)[2]. The reaction mixture was stirred at room temperature for 24 h before being diluted with water (3 mL) and acidified with 1N HCl to pH 6. The water solution was extracted with ethyl acetate and washed with brine. The crude product was purified by silica gel column chromatography using 0–13% methanol in dichloromethane.

2-((8,9-dimethylthieno[3,2-e][1,2,4]triazolo[4,3-c]pyrimidin-3-yl)thio)acetic acid (1)
White solid, 4.0 mg (42%). ^{1}H NMR (400 MHz, $DMSO_{d6}$) δ 9.16 (s, 1H), 7.59 (s, 1H), 4.00 (s, 2H), 2.63 (s, 3H), 2.54 (s, 3H); ^{13}C NMR (100 MHz, $DMSO_{d6}$) δ 169.7, 148.4, 147.1, 141.0, 135.5, 134.4, 127.1, 119.0, 37.2, 13.2, 12.9. Mass ESI $[M+H]^+$ = 294.98.

2-((8,9-dimethylthieno[3,2-e][1,2,4]triazolo[4,3-c]pyrimidin-3-yl)thio)-N-methylacetamide (2)
General procedure B was followed using **S4** and 2-bromo-N-methylacetamide. Yellow solid, 10 mg (40%). ^{1}H NMR (500 MHz, $CDCl_3$) δ 8.70 (s, 1H), 7.57 (s, 1H), 3.95 (s, 2H), 2.84 (d, J = 4.2 Hz, 2H), 2.70 (s, 3H), 2.56 (s, 3H); ^{13}C NMR (126 MHz, $CDCl_3$) δ 168.2, 149.4, 147.9, 141.6, 137.2, 131.2, 127.9, 119.7, 36.5, 26.8, 13.7, 13.3. Mass ESI $[M+H]^+$ = 307.97.

Isopropyl 2-((8,9-dimethylthieno[3,2-e][1,2,4]triazolo[4,3-c]pyrimidin-3-yl)thio)acetate (3)
General procedure B was followed using **S4** and isopropyl 2-bromoacetate. Yellow solid, 12 mg (45%). ^{1}H NMR (500 MHz, $CDCl_3$) δ 8.91 (s, 1H), 4.96 (sep, J = 5.0, 1H), 3.98 (s, 2H), 2.72 (s, 3H), 2.55 (s, 3H), 1.18 (s, 3H), 1.17 (s, 3H); ^{13}C NMR (126 MHz, $CDCl_3$) δ 167.9, 149.7, 148.1, 140.1, 136.8, 132.3, 128.1, 120.0, 70.3, 37.4, 21.8, 13.8, 13.5. Mass ESI $[M+H]^+$ = 337.03.

Tert-butyl 2-((8,9-dimethylthieno[3,2-e][1,2,4]triazolo[4,3-c]pyrimidin-3-yl)thio)acetate (4)
General procedure B was followed using **S4** and 1-bromo-3,3-dimethylbutan-2-one. Brown solid, 16 mg (55%). ^{1}H NMR (400 MHz, $CDCl_3$) δ 8.91 (s, 1H), 3.96 (s, 2H), 2.75 (s, 3H), 2.56 (s, 3H), 1.39 (s, 9H); ^{13}C NMR (100 MHz, $CDCl_3$) δ 167.4, 149.5, 148.1, 140.2, 136.7, 132.3, 128.1, 120.0, 83.2, 38.1, 28.0, 13.8, 13.4. Mass ESI $[M+H]^+$ = 351.08.

2-((8,9-dimethylthieno[3,2-e][1,2,4]triazolo[4,3-c]pyrimidin-3-yl)thio)ethan-1-ol (5)
General procedure B was followed using **S4** and 2-bromoethan-1-ol. Light yellow solid, 12 mg (51%). ^{1}H NMR (400 MHz, $CDCl_3$) δ 8.74 (s, 1H), 4.06 (t, J = 4.0, 2H), 3.47 (t, J = 4.0, 2H), 2.65 (s, 3H), 2.54 (s, 3H); ^{13}C NMR (100 MHz, $CDCl_3$) δ 149.5, 147.7, 141.7, 137.0, 131.8, 128.1, 119.8, 62.2, 37.4, 13.8, 13.4. Mass ESI $[M+H]^+$ = 281.00.

Methyl 2-((8,9-dimethylthieno[3,2-e][1,2,4]triazolo[4,3-c]pyrimidin-3-yl)thio)-2-methylpropanoate (6)
General procedure B was followed using **S4** and methyl 2-bromo-2-methylpropanoate. Off white solid, 13 mg (47%). ^{1}H NMR (400 MHz, $CDCl_3$) δ 8.89 (s, 1H), 3.58 (s, 3H), 2.77 (s, 3H), 2.57 (s, 3H), 1.68 (s, 6H); ^{13}C NMR (100 MHz, $CDCl_3$) δ 173.5, 150.0, 148.6, 138.2, 136.9, 132.5, 128.1, 120.1, 54.0, 53.1, 26.3, 13.8, 13.4. Mass ESI $[M+H]^+$ = 337.06.

3-(((2H-tetrazol-5-yl)methyl)thio)-8,9-dimethylthieno[3,2-e][1,2,4]triazolo[4,3-c]pyrimidine (7)
Following general procedure B, using **S4** and 2-bromoacetonitrile, 11 mg **S5** was obtained as a pale solid (68%).

A mixture of **S5** (11 mg, 0.04 mmol), NaN_3 (5.2mg, 0.08 mmol) and triethylamine hydrochloride (8.3 mg, 0.06 mmol) in DMF (0.5 mL) was heated to 110 °C for 3 hours. DMF was removed under vacuum and the residue was purified by silica-gel column chromatography using 0-10 % methanol in dichloromethane. Off white solid, 6 mg (47%). ^{1}H NMR (400 MHz, $DMSO_{d6}$) δ 9.50 (s, 1H), 4.88 (s, 2H), 2.54 (s, 3H), 2.53 (s, 3H); ^{13}C NMR (100 MHz, $DMSO_{d6}$) δ 163.9, 154.3, 152.4, 149.1, 136.2, 135.7, 126.3, 120.0, 23.4, 13.3, 12.6. Mass ESI $[M+H]^+$ = 319.03.

C. Syntheses of compounds 8–10
Methyl 2-((5,8,9-trimethylthieno[3,2-e][1,2,4]triazolo[4,3-c]pyrimidin-3-yl)thio)acetate (8)

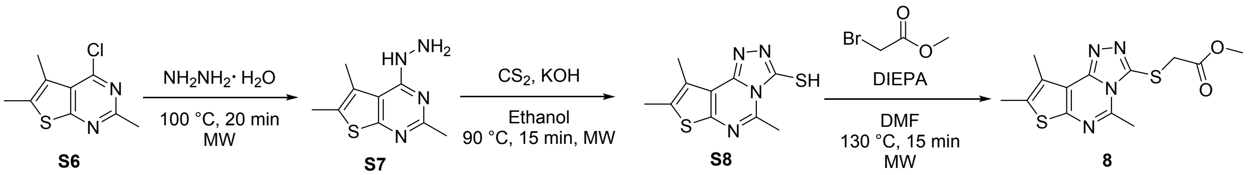

Following Procedure A (steps 2 and 3), compound **S6** (50 mg, 23 mmol) was converted into 22 mg compound **S8**. Yellow solid (yield over two steps: 37%). Then, Procedure B was followed using **S8** and methyl 2-bromoacetate to synthesize compound **8**. Off white solid, 15 mg (58%). ^{1}H NMR (400 MHz, $CDCl_3$) δ 4.25 (s, 2H), 3.78 (s, 3H), 3.14 (s, 3H), 2.66 (s, 3H), 2.49 (s, 3H); ^{13}C NMR (100 MHz, $CDCl_3$) δ 168.7, 149.5, 149.2, 142.5, 141.9, 134.7, 128.0, 119.0, 53.2, 36.1, 23.1, 13.6, 13.4. Mass ESI $[M+H]^+$ = 323.04.

Methyl 2-((8,9-dimethylimidazo[1,5-c]thieno[3,2-e]pyrimidin-3-yl)thio)acetate (9)

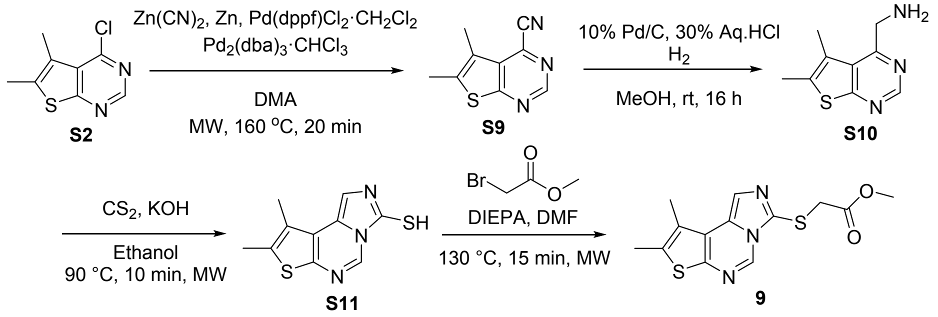

Step 1: In a 5 mL microwave vial was added **S2** (100 mg, 0.5 mmol), $Zn(CN)_2$ (36 mg, 0.33 mmol), zinc powder (4 mg, 0.06 mmol), $Pd(dppf)Cl_2\cdot CH_2Cl_2$ (5 mg, 0.005 mmol), $Pd_2(dba)_3\cdot CHCl_3$ (5.2 mg, 0.005 mmol) and DMA (1 mL). The vial was evacuated and refilled with N_2. The reaction mixture was heated under microwave irradiation for 20 min. The reaction was then quenched by adding ice-cold water, and then extracted with ethyl acetate. The organic layer was dried with Na_2SO_4 and concentrated in vacuo. The residue was purified by silica gel column chromatography using 0–8% ethyl acetate in hexanes to give **S9** as a brown solid, 87 mg (92%).

Step 2: Compound **S9** (87 mg, 0.46 mmol) in methanol (10 mL) was added 10% Pd/C (10 mg) and 30% HCl in water (0.2 mL) in a round-bottom flask. The flask was evacuated and refilled with hydrogen. The reaction mixture was stirred overnight at room temperature. Pd/C was removed by filtration and the filtrate was concentrated in vacuo. Brown solid, 62 mg (70 %).

Steps 3 and 4: Procedures A and B were followed to obtain compound **9** as a pale brown solid, 11 mg (43%). ^{1}H NMR (500 MHz, $CDCl_3$) δ 8.96 (s, 1H), 7.66 (s, 1H), 3.80 (s, 2H), 3.65 (s, 3H), 2.51 (s, 3H), 2.44 (s, 3H); ^{13}C NMR (126 MHz, $CDCl_3$) δ 169.4, 143.4, 135.2, 132.7, 129.6, 129.1, 126.5, 122.1, 118.4, 52.9, 37.3, 13.6, 13.1. Mass ESI $[M+H]^+$ = 308.00.

Methyl 2-((8,9-dimethylthieno[3,2-c][1,2,4]triazolo[4,3-a]pyridin-3-yl)thio)acetate (10)

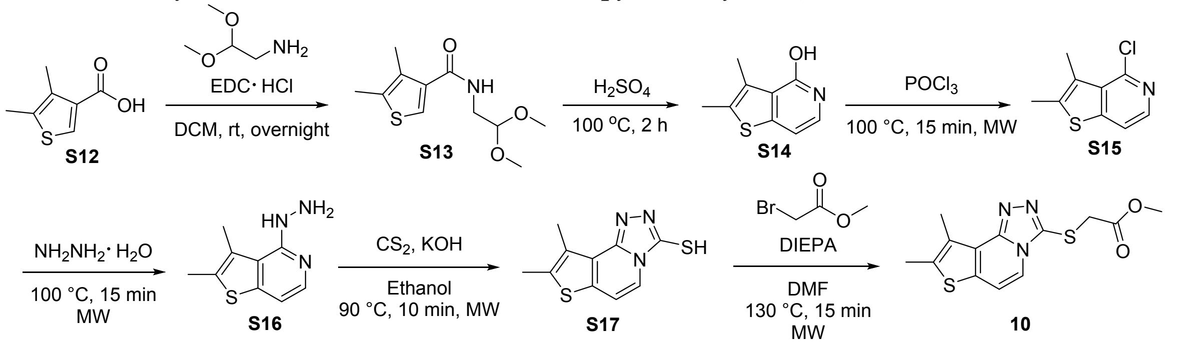

Step 1: 4,5-dimethylthiophene-3-carboxylic acid (250 mg, 1.6 mmol) in dichloromethane (15 mL) was added EDC·HCl (370 mg, 1.93 mmol) at 0 °C and stirred for 5 min. Then to the reaction mixture was added 2,2-dimethoxyethan-1-amine (0.22 mL, 1.93 mmol) in dichloromethane (1 mL) dropwise. The mixture was stirred at room temperature for overnight. Water was added and the mixture was extracted with dichloromethane, washed with brine and dried with Na_2SO_4. The residue was purified by silica gel column chromatography using 0–20% ethyl acetate in hexanes to obtain compound **S13**. Colorless oil, 230 mg (59%).

Step 2: A solution of **S13** (100 mg, 0.41 mmol) in concentrated H_2SO_4 (1 mL) was heated at 100°C for 2 h. The mixture was consecutively cooled to room temperature, and 6 M NaOH was added carefully to adjust the pH to 10. The aqueous solution was extracted with ethyl acetate (5 × 15 mL), the combined organic layers were dried over anhydrous Na_2SO_4, filtered, and concentrated in vacuo. The residue was purified by silica gel column chromatography using 0–25% ethyl acetate in hexanes to give compound **S14**. Pale brown solid, 50 mg (68%).

Steps 3 to 6: Procedures A and B were followed to give the final product **10** as an off-white solid, 11mg (45%). ^{1}H NMR (500 MHz, $CDCl_3$) δ 8.03 (d, J = 7.5 Hz, 1H), 7.23 (d, J = 7.5 Hz, 1H), 3.95 (s, 2H), 3.66 (s, 3H), 2.79 (s, 3H), 2.54 (s, 3H); ^{13}C NMR (126 MHz, $CDCl_3$) δ 169.1, 148.6, 140.2, 137.0, 134.6, 130.4, 126.2, 117.7, 110.2, 52.9, 36.8, 13.7, 13.6. Mass ESI $[M+H]^+$ = 307.97.

References

1. Raghu Prasad M, Raghuram Rao A, Shanthan Rao P, Rajan KS, Meena S, Madhavi K. Synthesis and adenosine receptor binding studies of some novel triazolothienopyrimidines. *Eur J Med Chem*. 2008;43(3):614-620. doi:10.1016/j.ejmech.2007.05.001

2. Pinto A, Serra I, Romano D, et al. Preparation of Sterically Demanding 2,2-Disubstituted-2-Hydroxy Acids by Enzymatic Hydrolysis. *Catalysts*. 2019;9(2). doi:10.3390/catal9020113

NMR Spectra

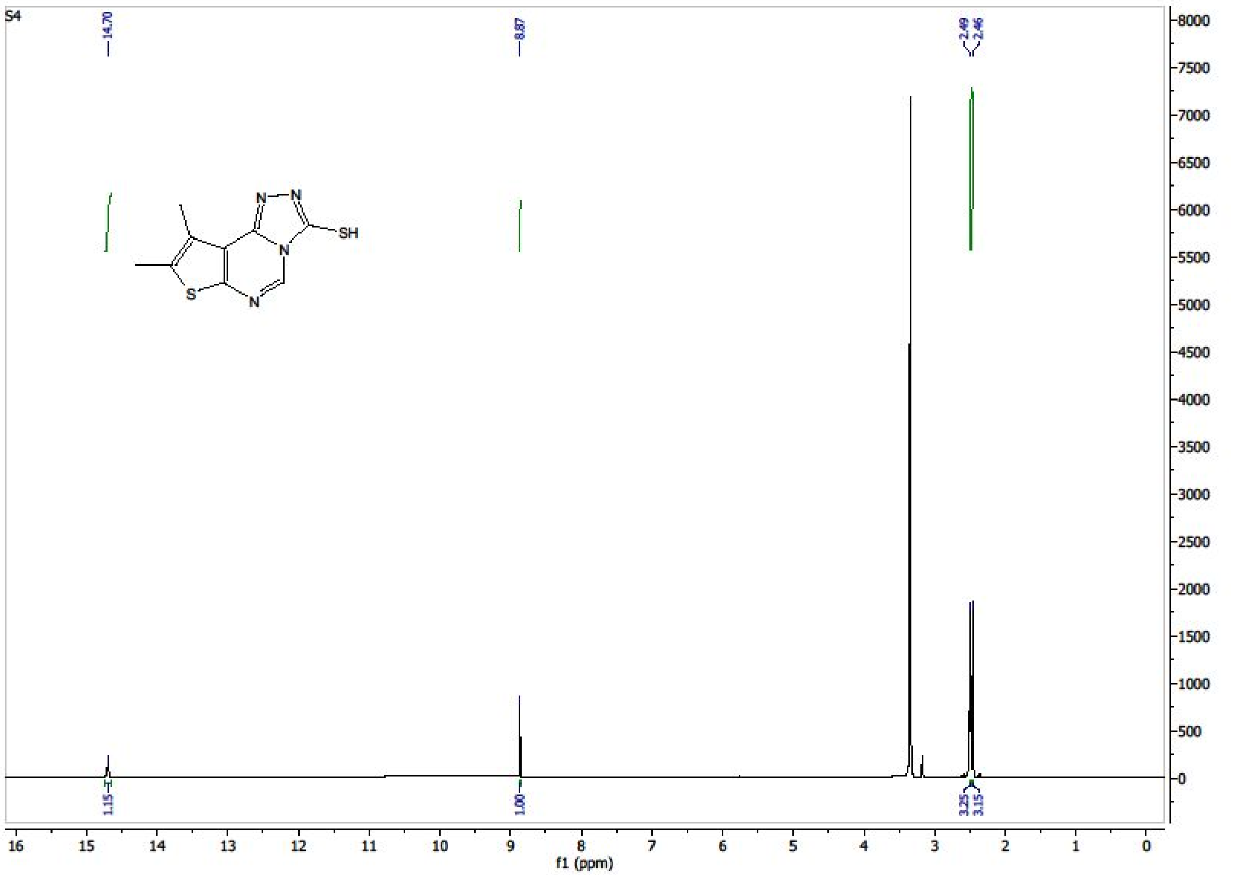

S4
14.70
8.87
2.49
2.46
SH
N
S
1.15
1.00
3.25
3.15
f1 (ppm)

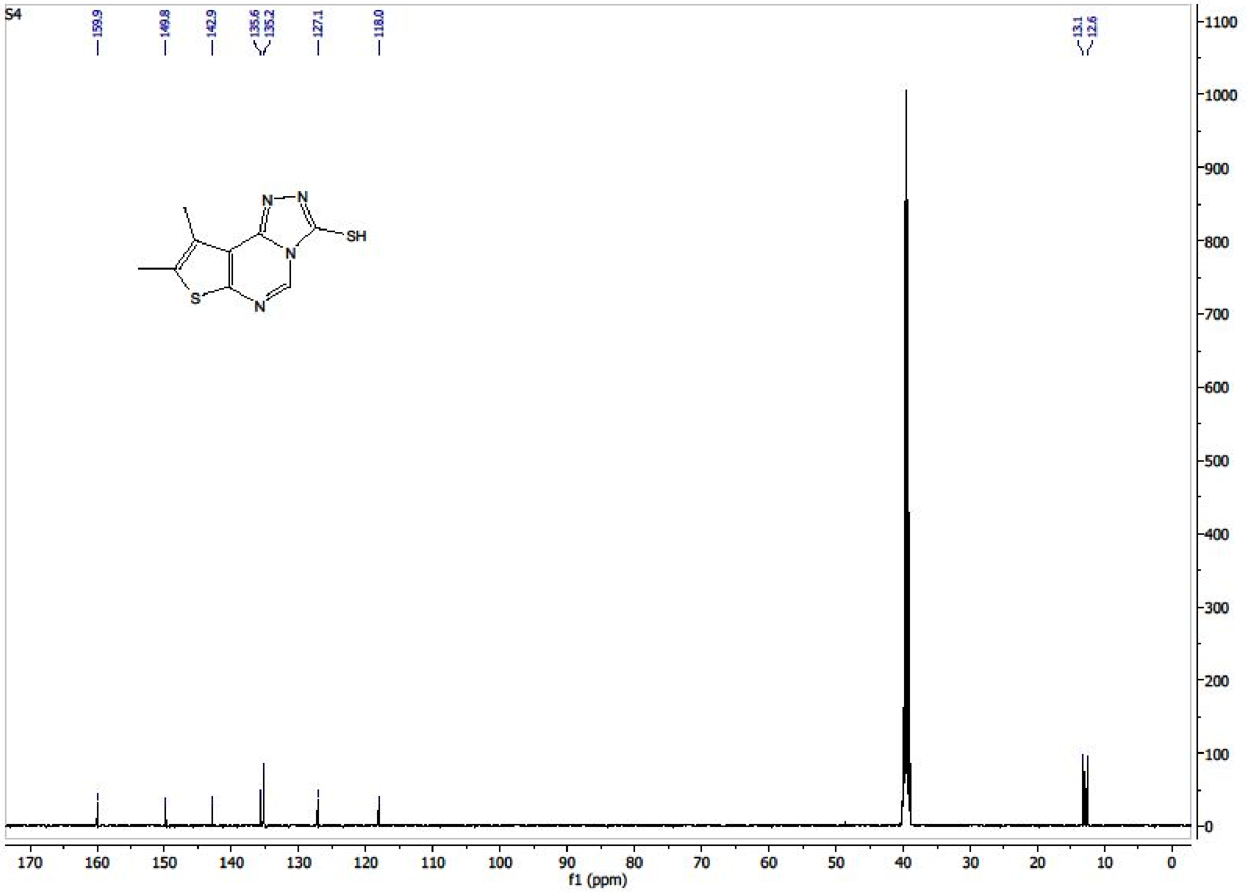

S4
159.9
149.8
142.9
135.6
135.2
127.1
118.0
13.1
12.6
SH
N
S
f1 (ppm)

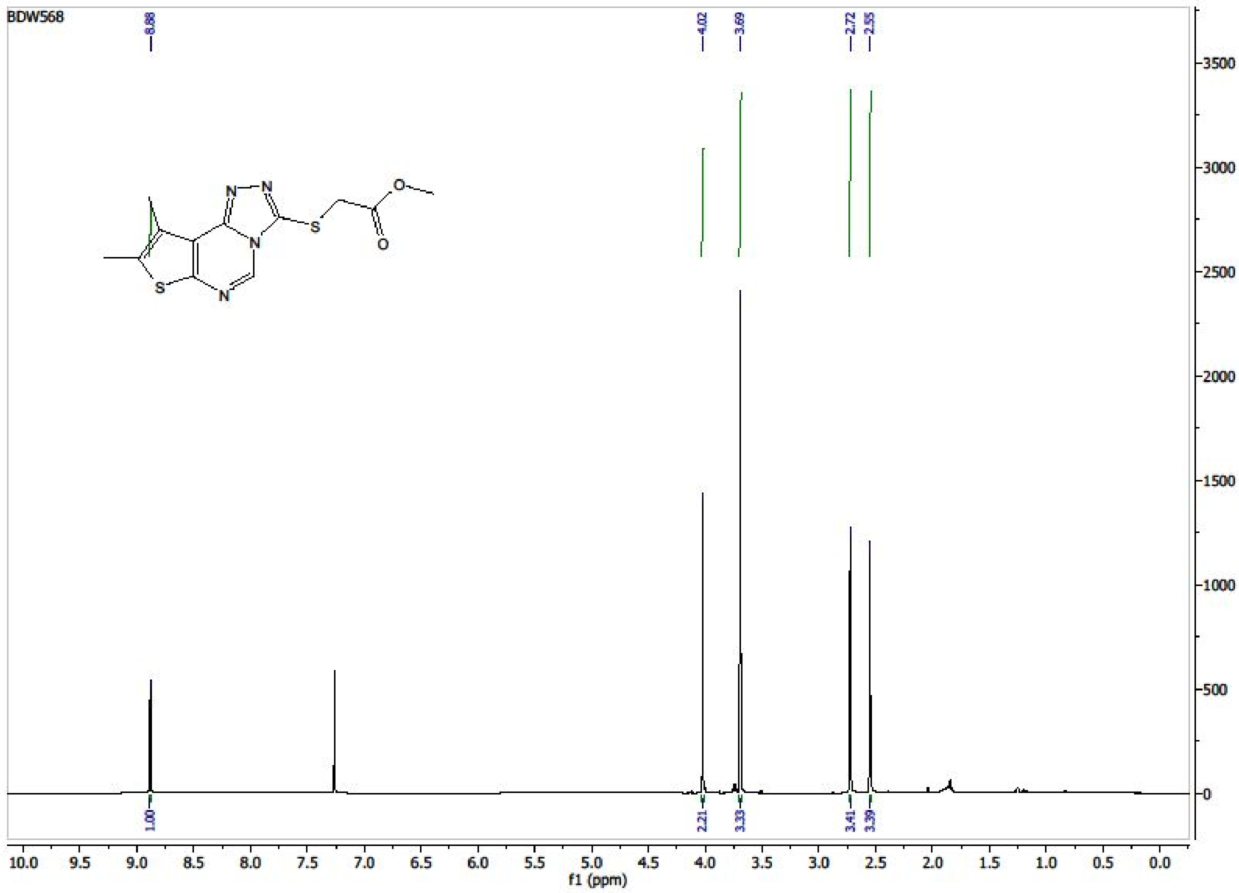
BDW568
8.88
4.02
3.69
2.72
2.55
1.00
2.21
3.33
3.41
3.39
f1 (ppm)

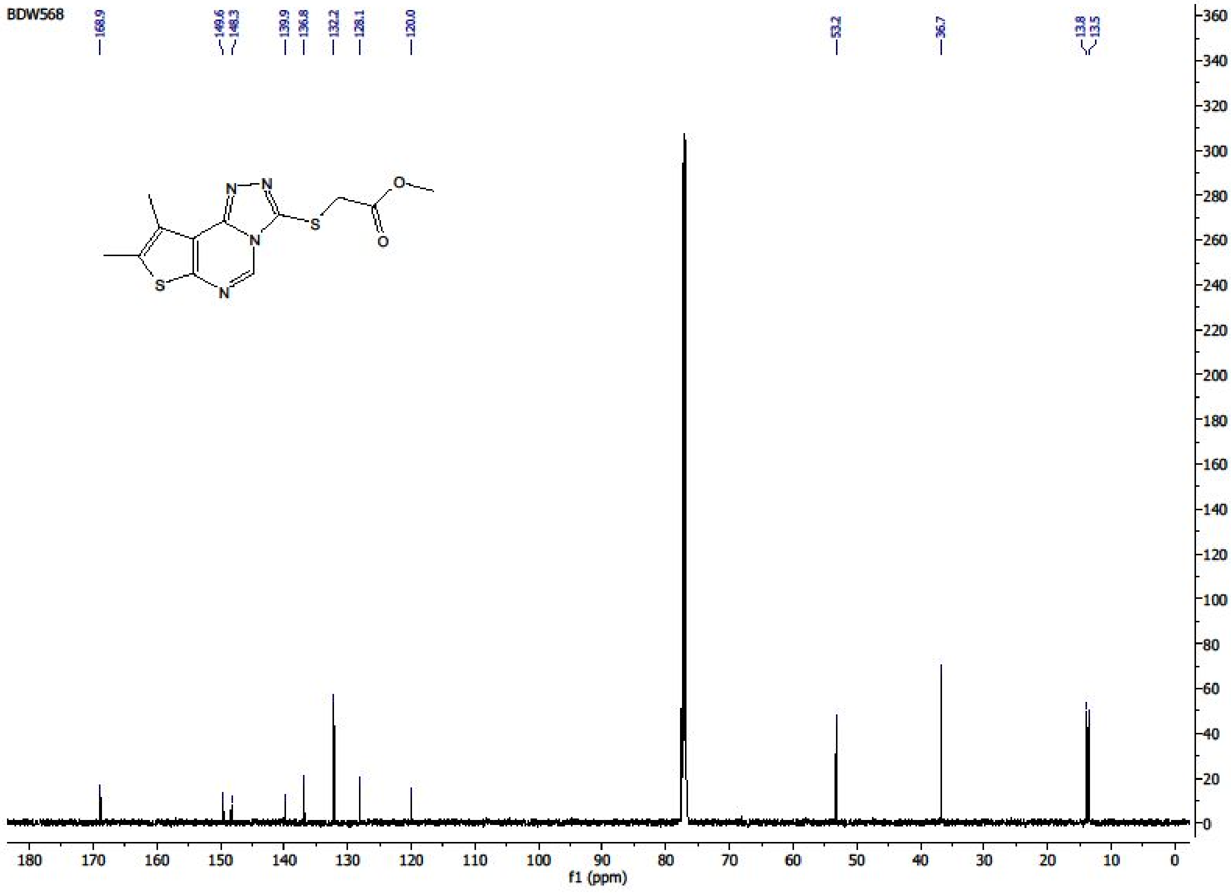
BDW568
168.9
149.6
148.3
139.9
136.8
132.2
128.1
120.0
53.2
36.7
13.8
13.5
f1 (ppm)

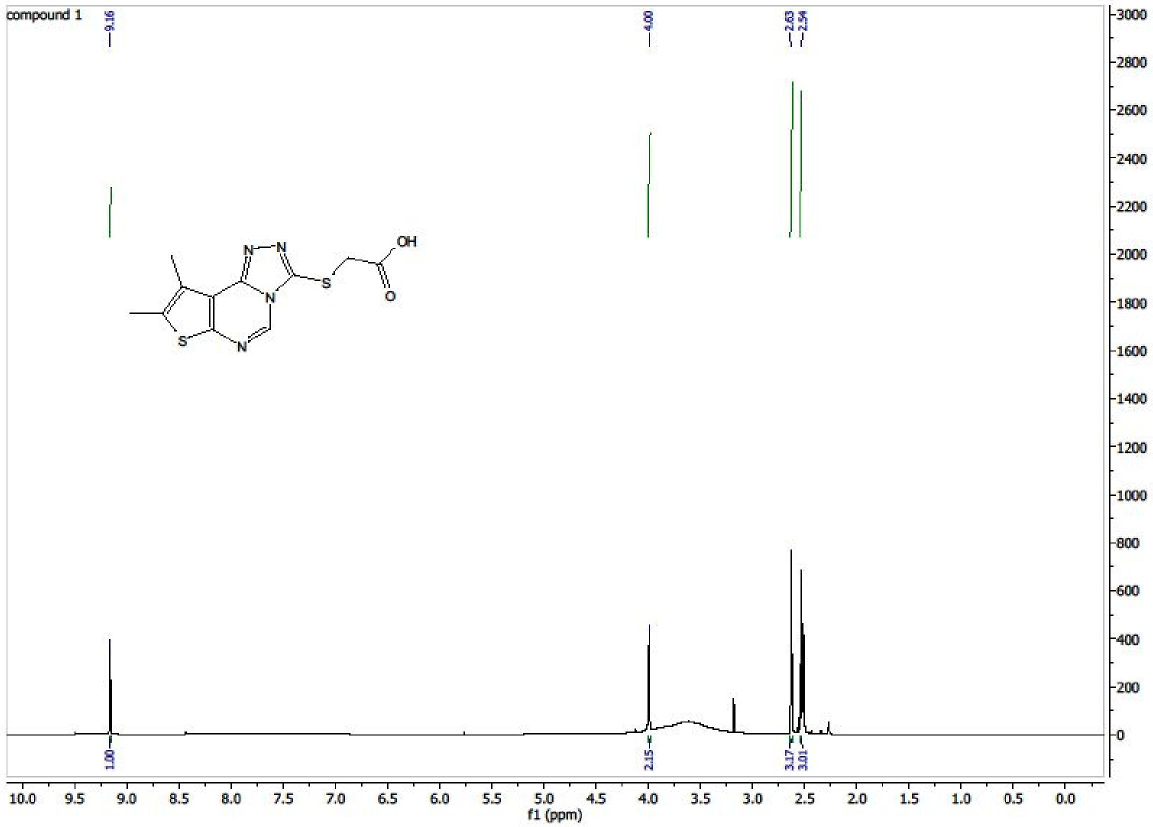

compound 1
9.16
4.00
2.63
2.54
1.00
2.15
3.17
3.01
f1 (ppm)
OH
O
S
N

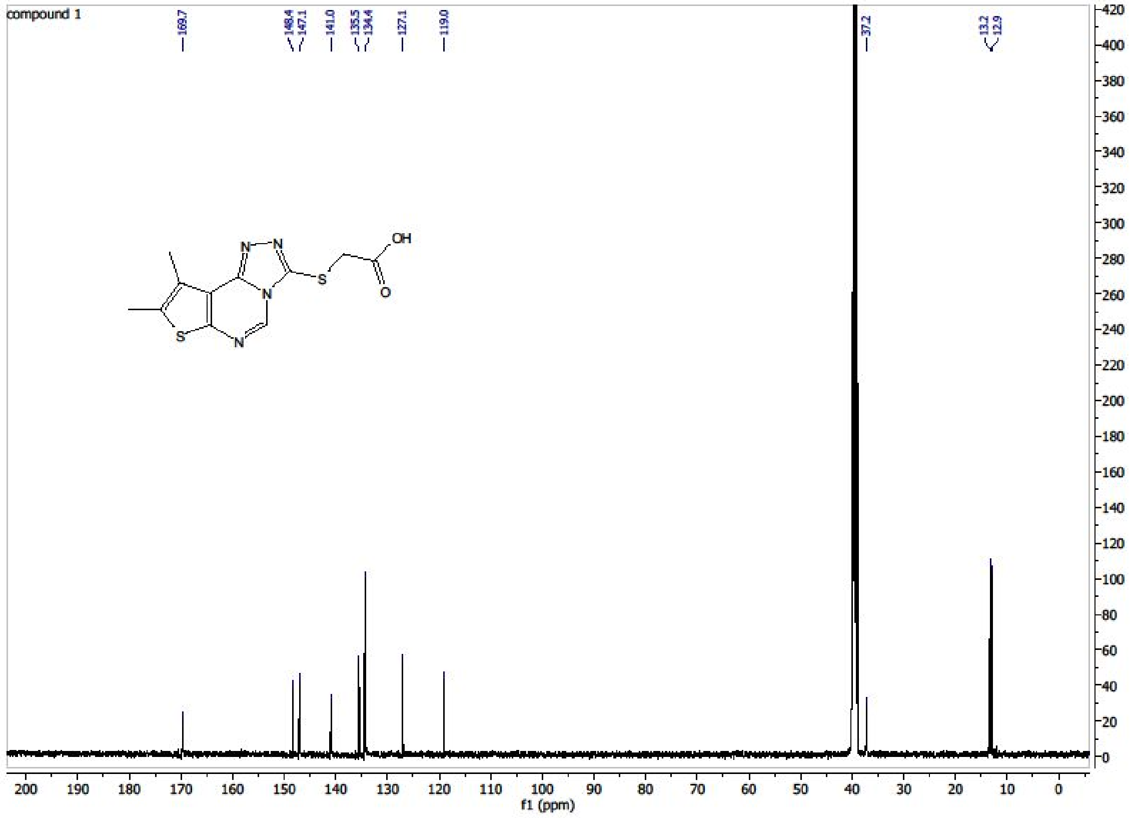

compound 1
169.7
148.4
147.1
141.0
135.5
134.4
127.1
119.0
37.2
13.2
12.9
f1 (ppm)
OH
O
S
N

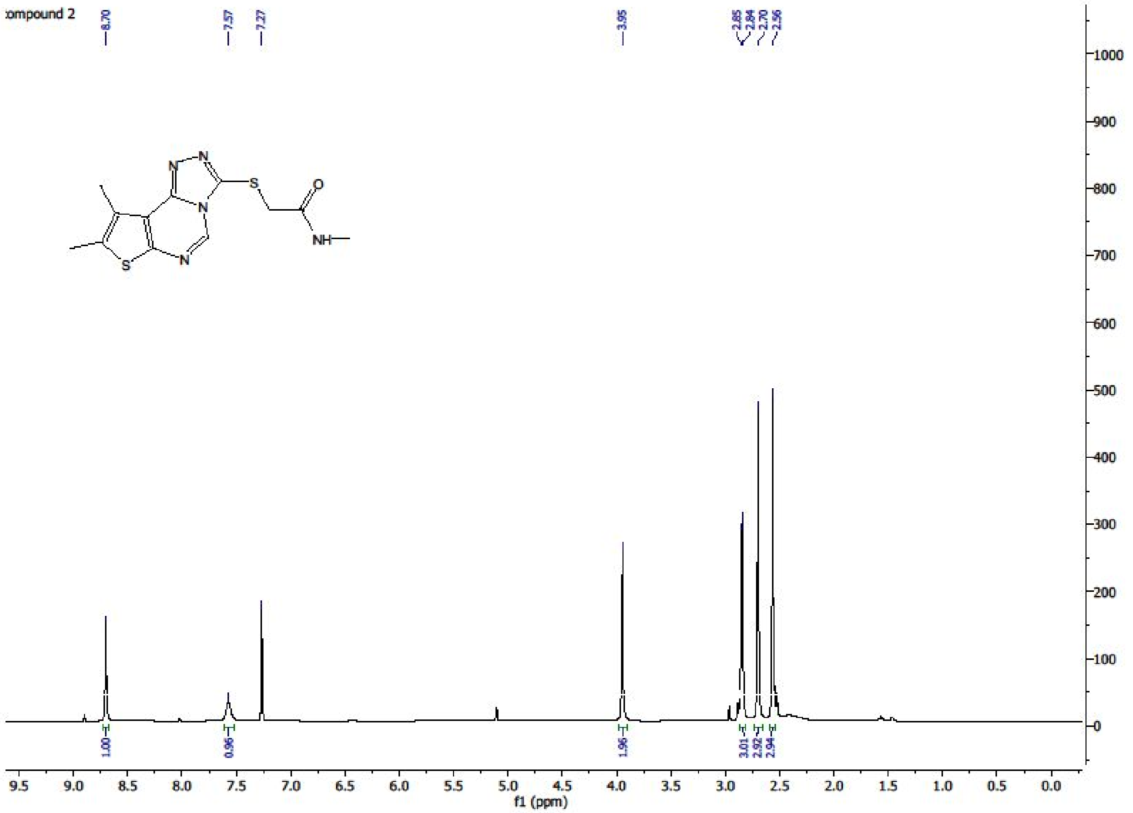
compound 2
8.70
7.57
7.27
3.95
2.85
2.84
2.70
2.56
1.00
0.96
1.96
3.01
2.92
2.94
f1 (ppm)

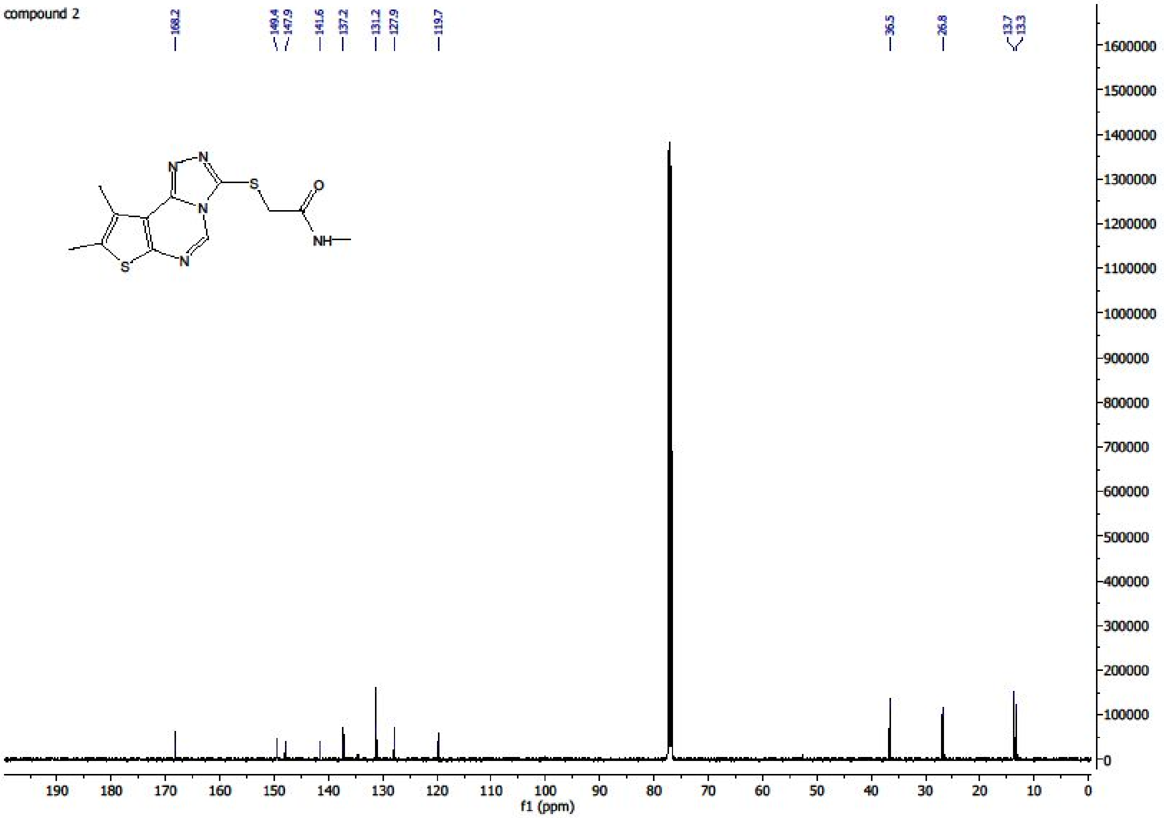
compound 2
168.2
149.4
147.9
141.6
137.2
131.2
127.9
119.7
36.5
26.8
13.7
13.3
f1 (ppm)

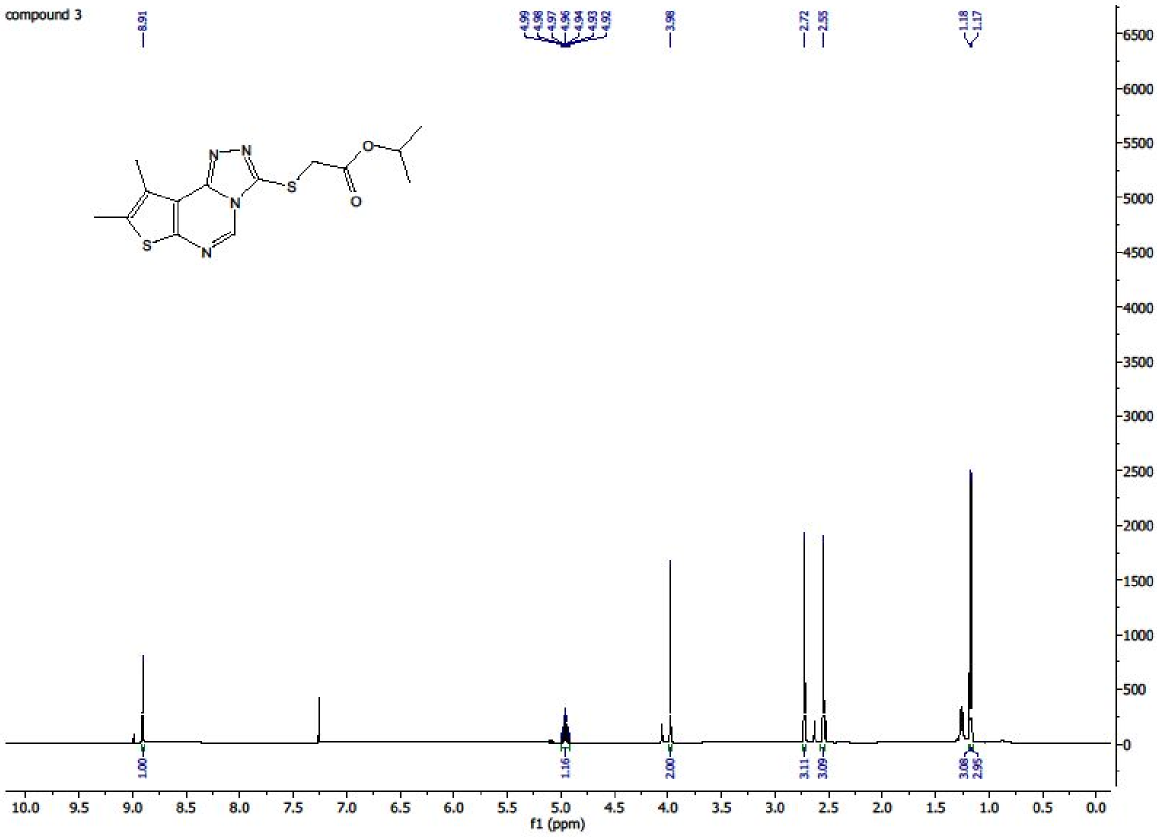
compound 3
f1 (ppm)

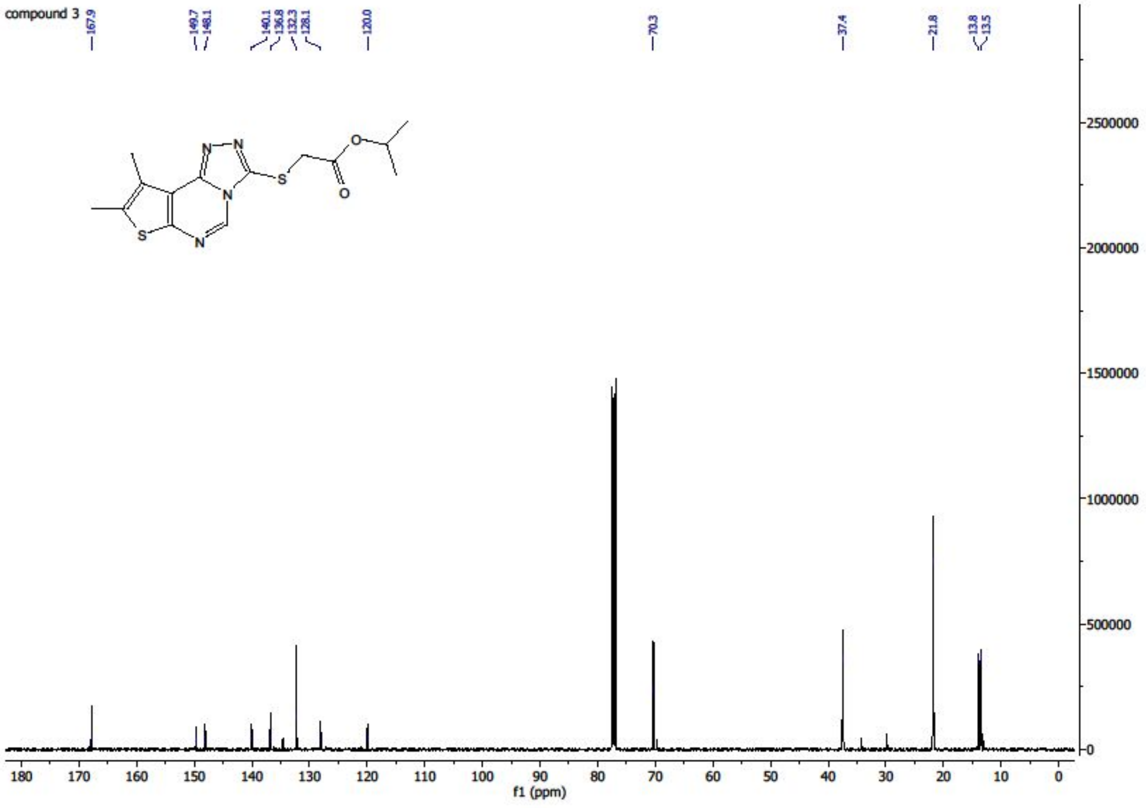
compound 3
f1 (ppm)

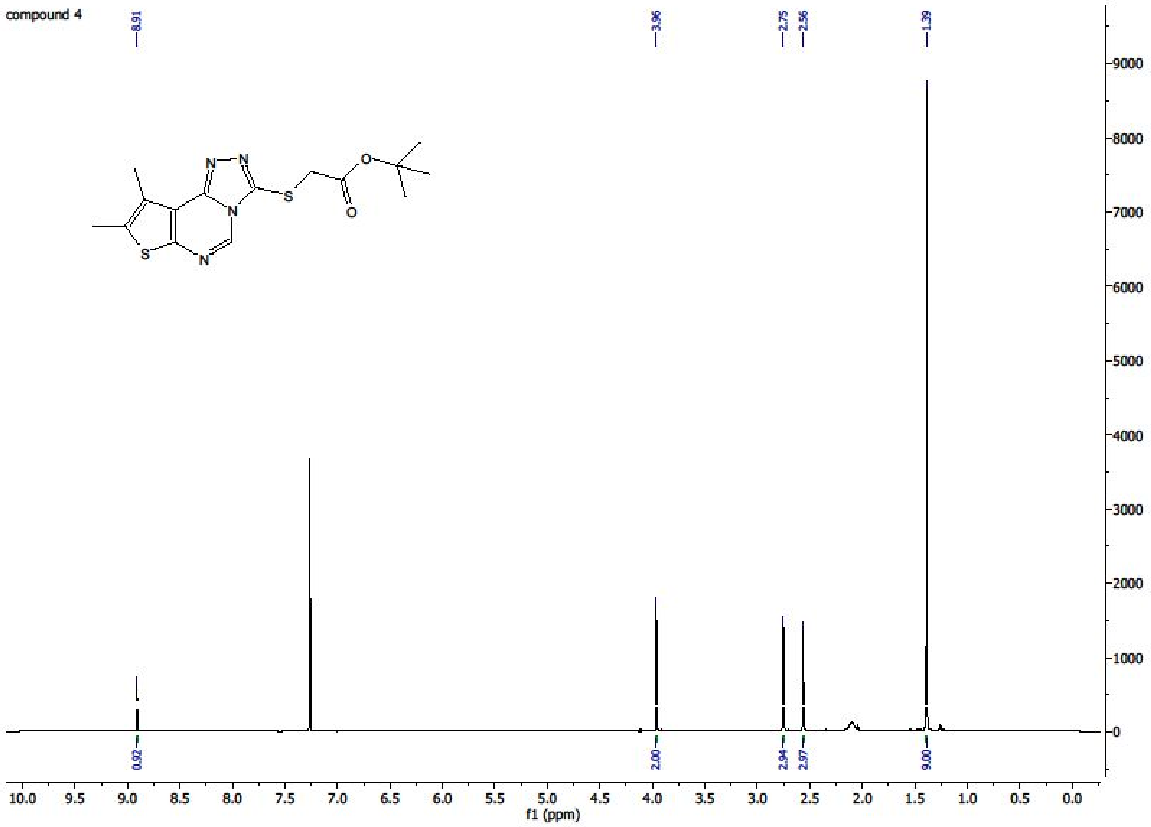
compound 4
8.91
3.96
2.75
2.56
1.39
0.92
2.00
2.94
2.97
9.00
f1 (ppm)

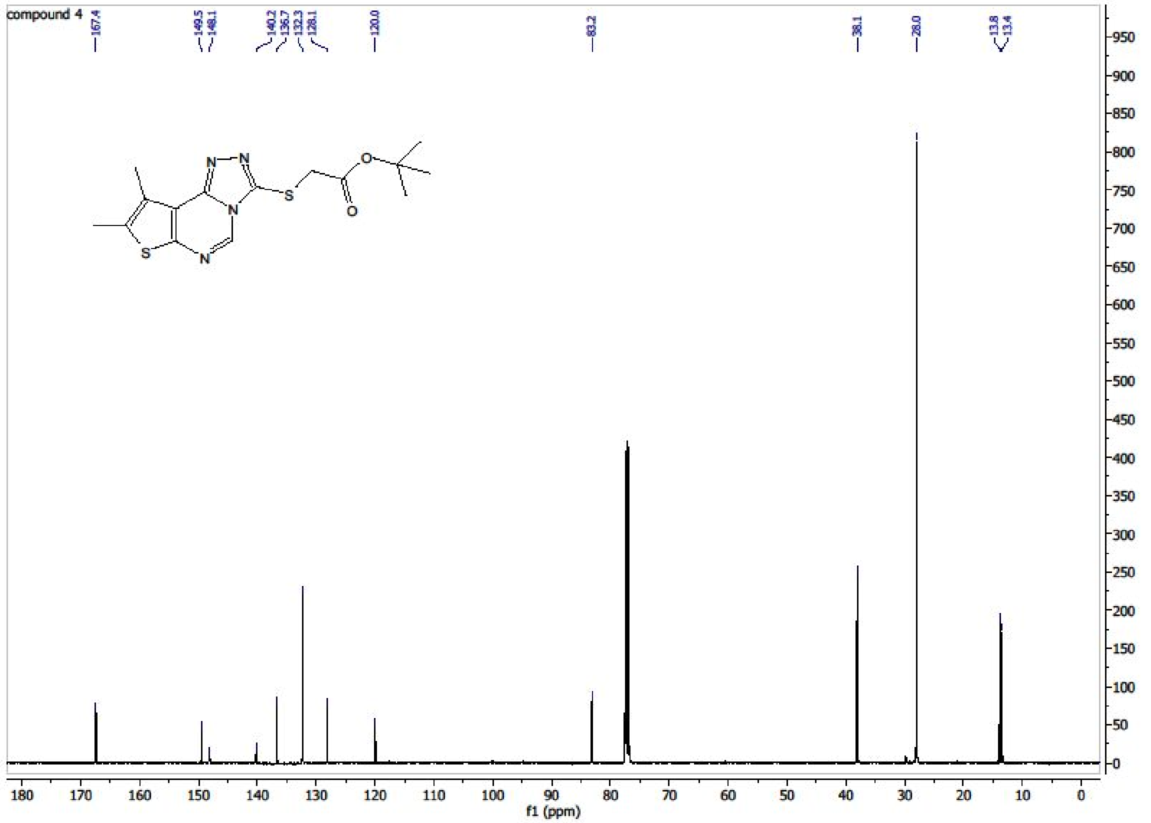
compound 4
167.4
149.5
148.1
140.2
136.7
132.3
128.1
120.0
83.2
38.1
28.0
13.8
13.4
f1 (ppm)

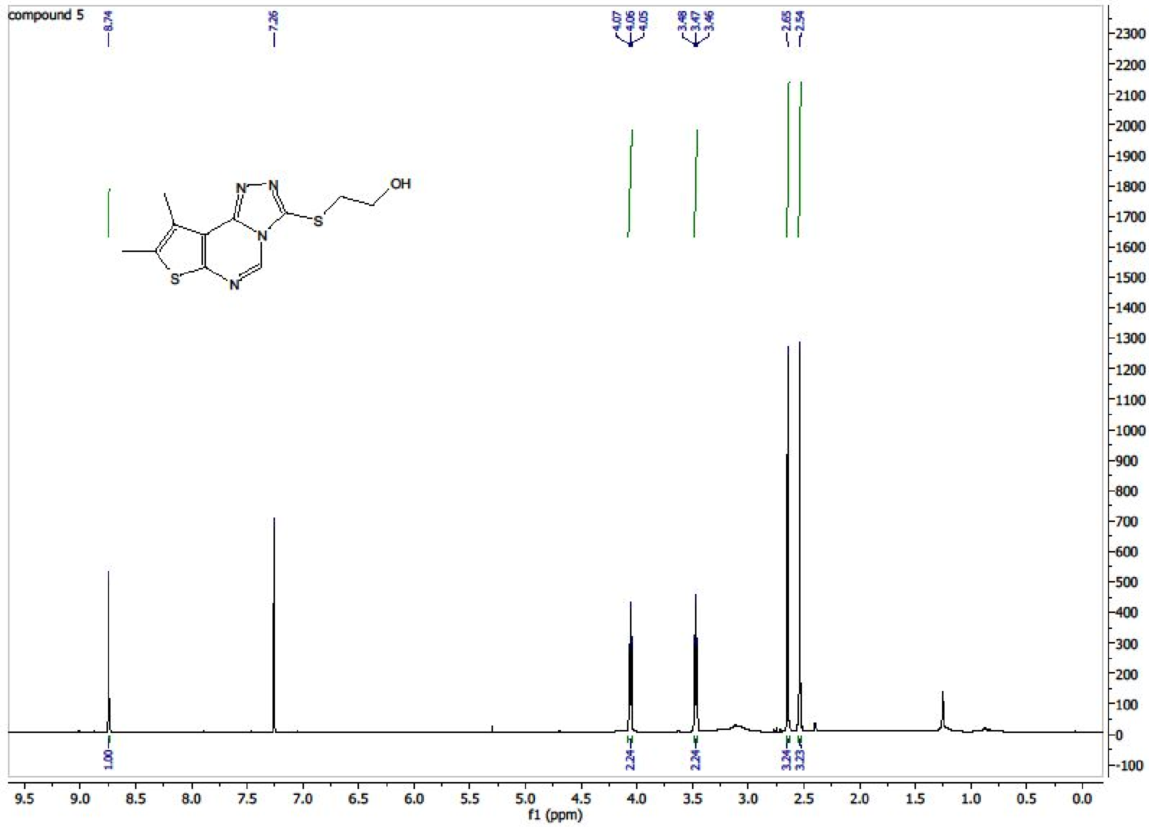
compound 5
8.74
7.26
4.07
4.06
4.05
3.48
3.47
3.46
2.65
2.54
1.00
2.24
2.24
3.24
3.23
f1 (ppm)

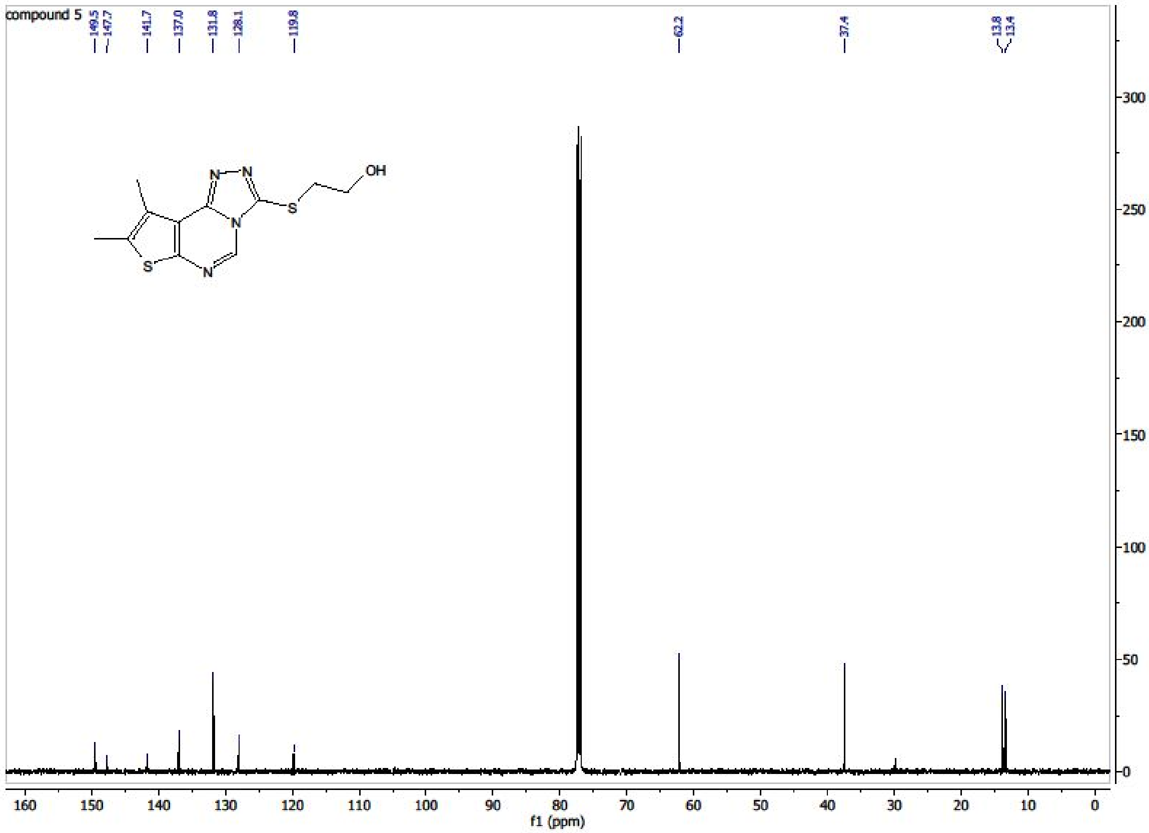
compound 5
149.5
147.7
141.7
137.0
131.8
128.1
119.8
62.2
37.4
13.8
13.4
f1 (ppm)

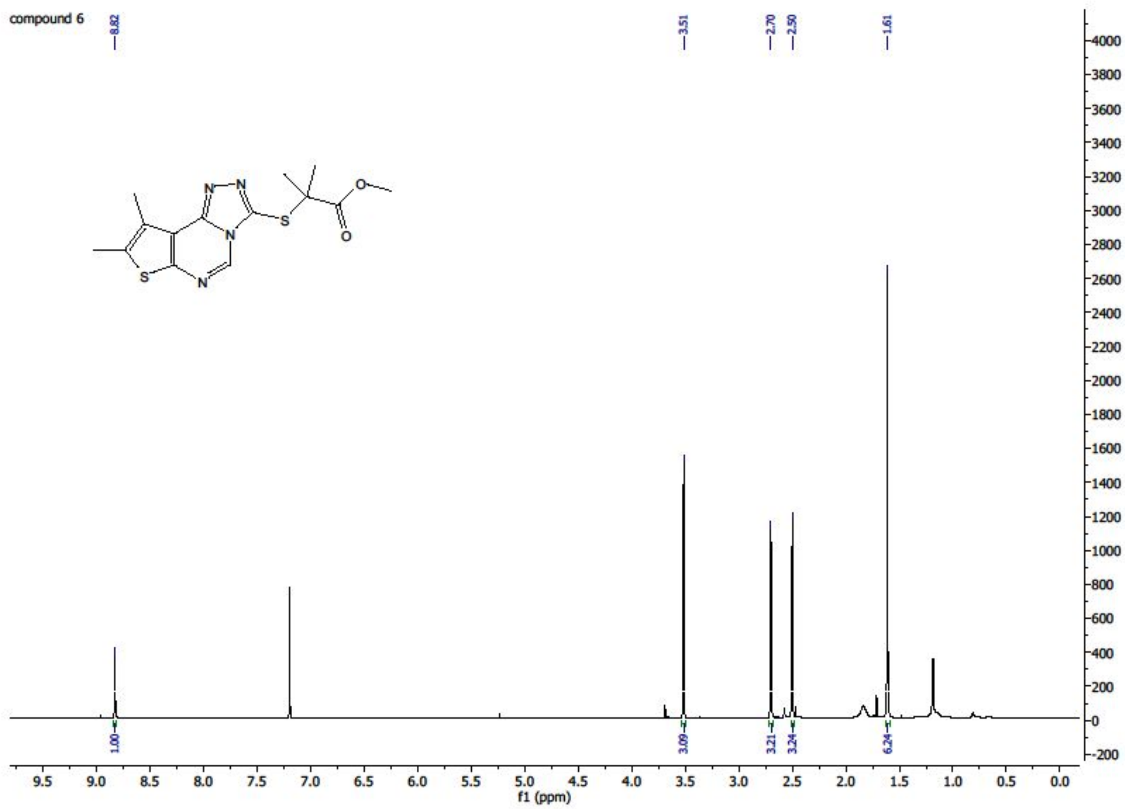
compound 6
8.82
3.51
2.70
2.50
1.61
1.00
3.09
3.21
3.24
6.24
f1 (ppm)

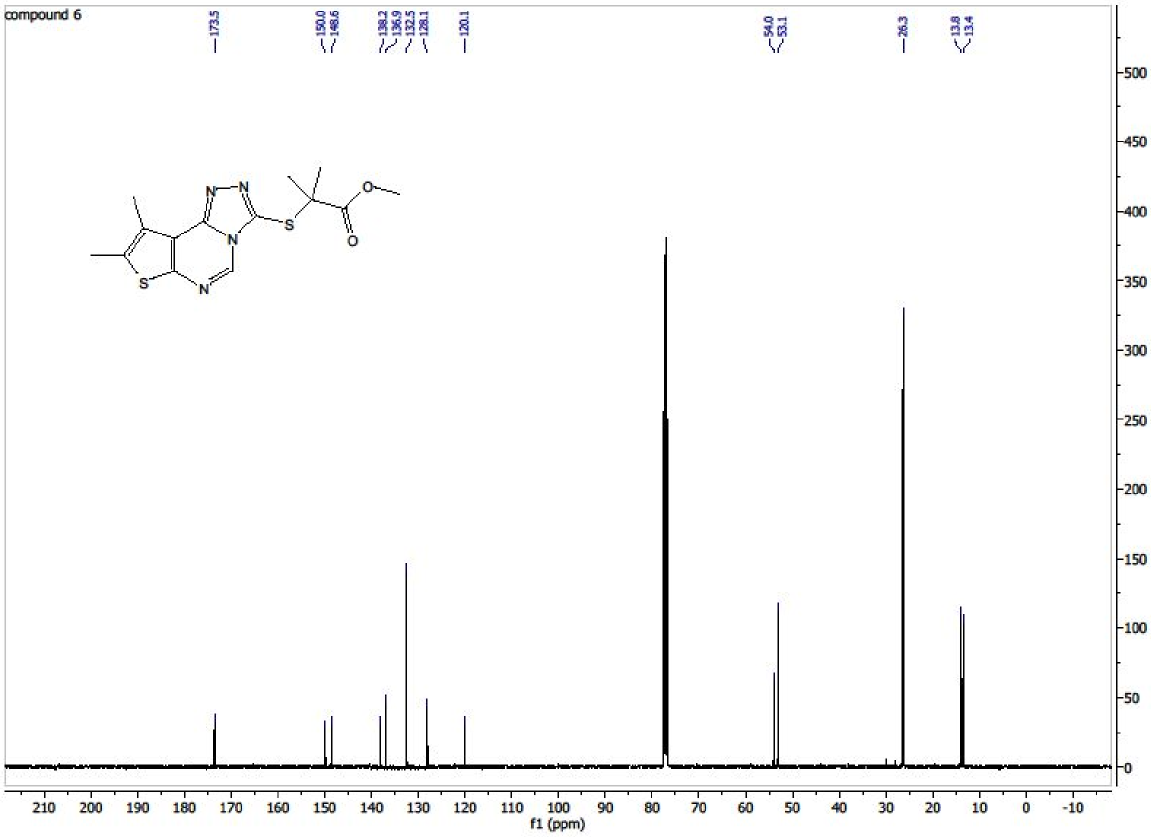
compound 6
173.5
150.0
148.6
138.2
136.9
132.5
128.1
120.1
54.0
53.1
26.3
13.8
13.4
f1 (ppm)

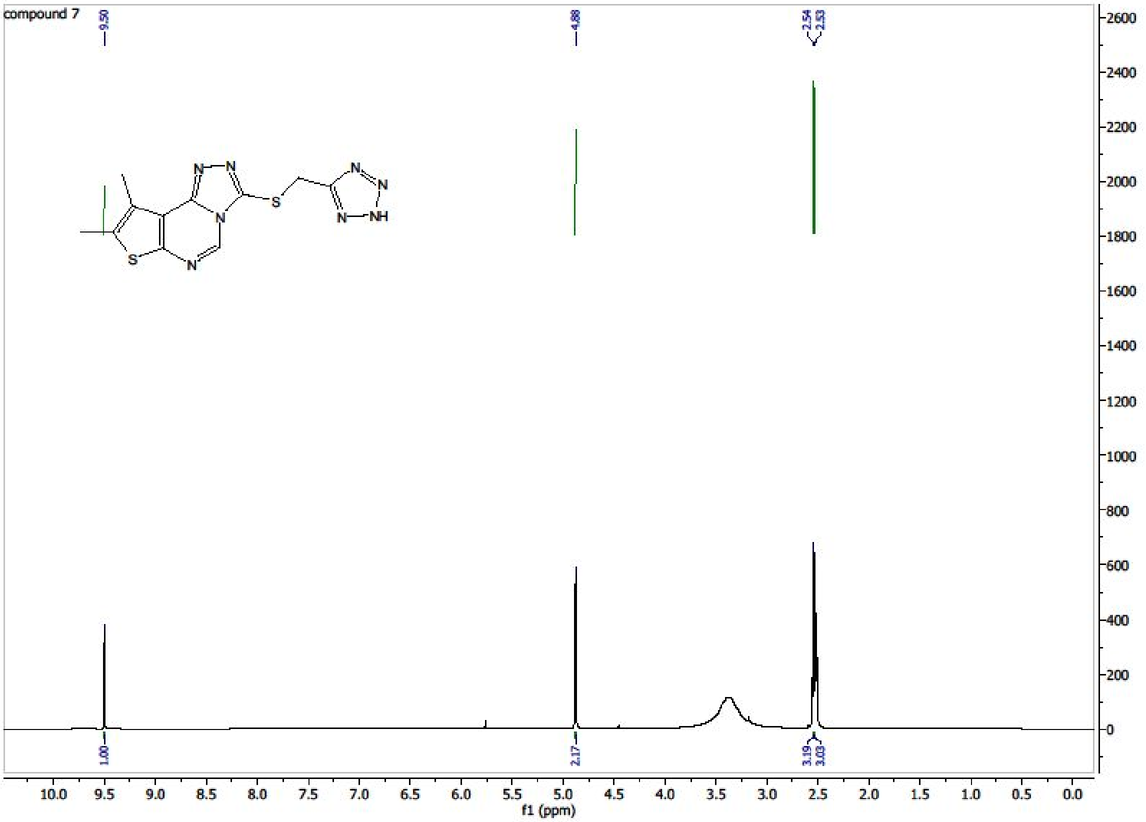
compound 7
9.50
4.88
2.54
2.53
N
N
S
N
N
N
NH
S
N
1.00
2.17
3.19
3.03
f1 (ppm)

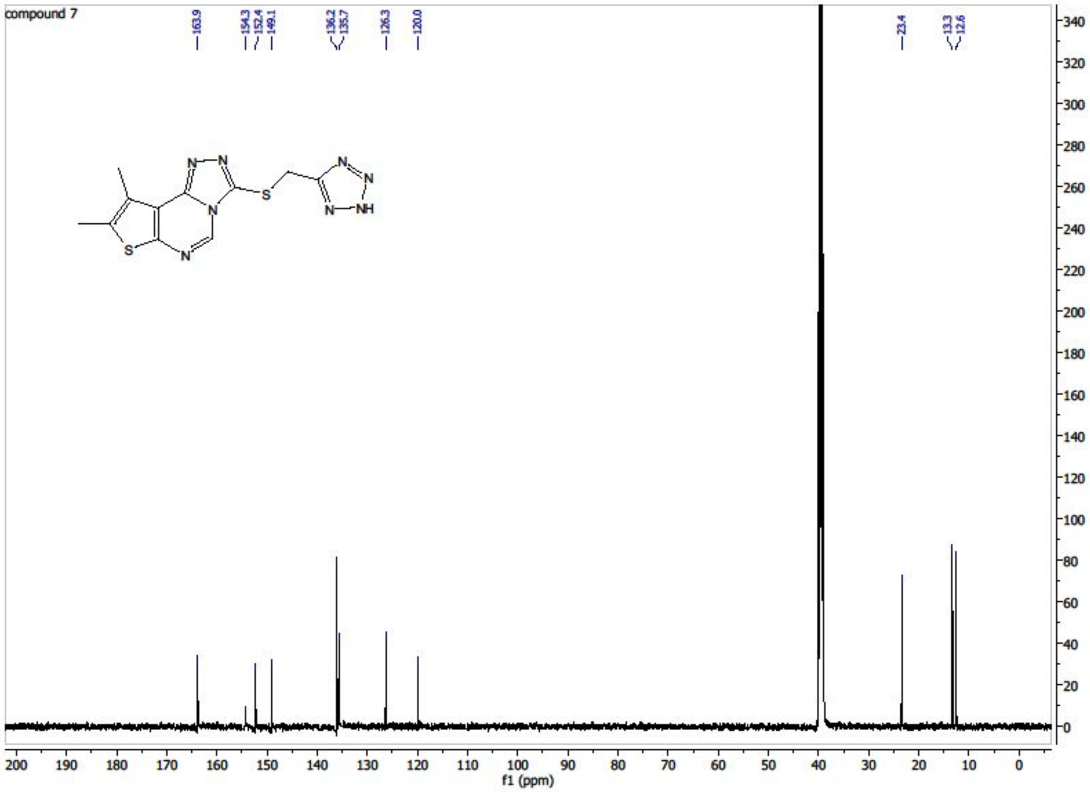
compound 7
163.9
154.3
152.4
149.1
136.2
135.7
126.3
120.0
23.4
13.3
12.6
N
N
S
N
N
N
NH
S
N
f1 (ppm)

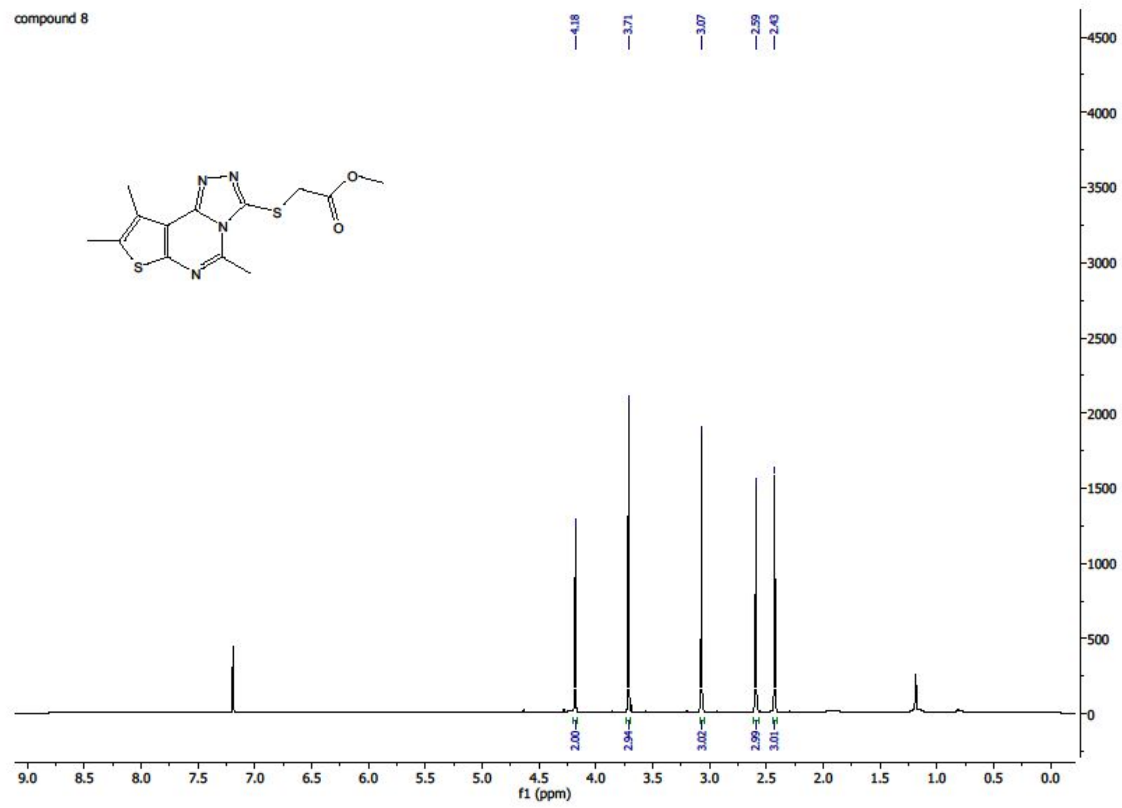
compound 8
4.18
3.71
3.07
2.59
2.43
2.00
2.94
3.02
2.99
3.01
f1 (ppm)

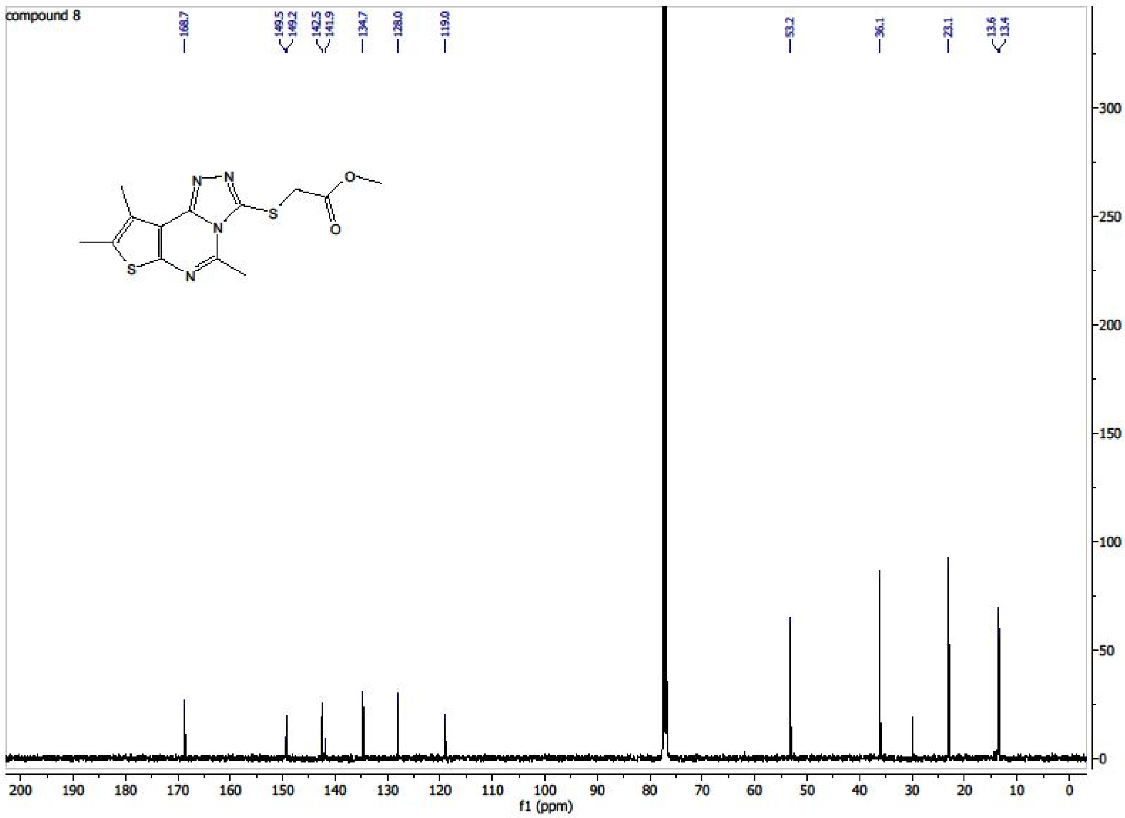
compound 8
168.7
149.5
149.2
142.5
141.9
134.7
128.0
119.0
53.2
36.1
23.1
13.6
13.4
f1 (ppm)

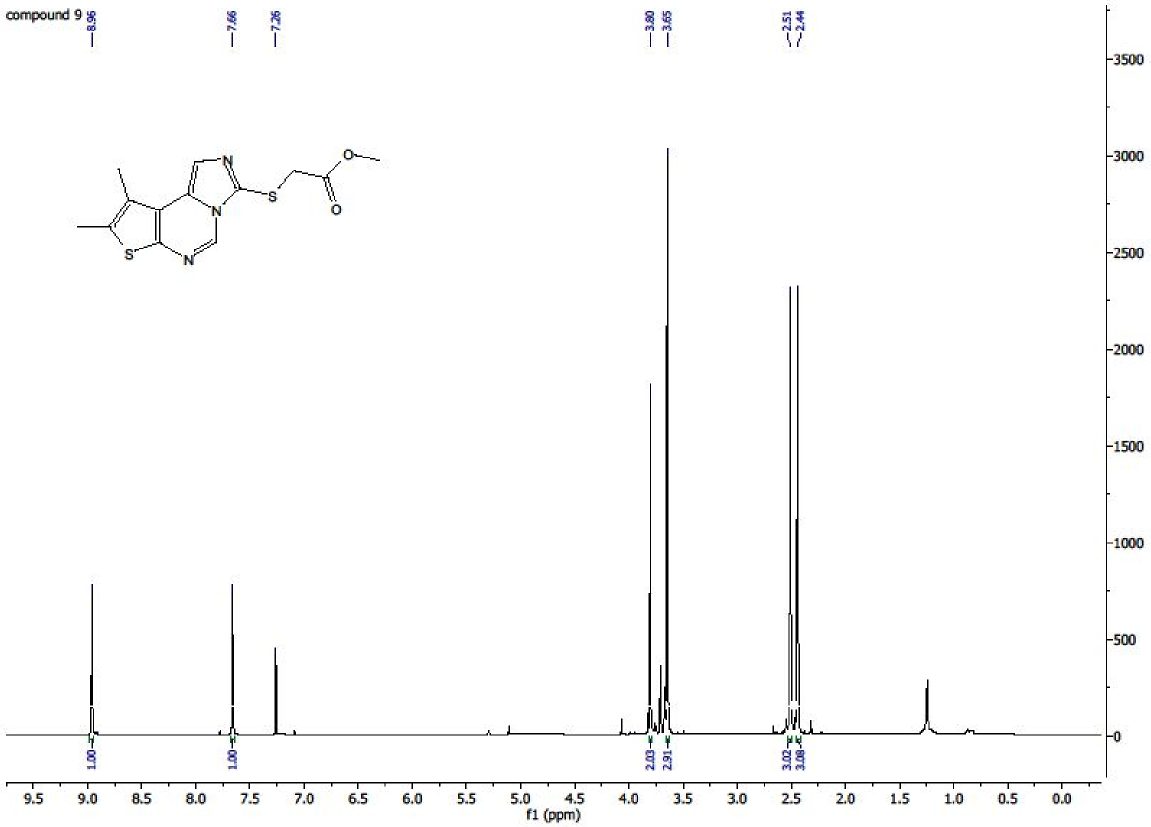
compound 9
8.96
7.66
7.26
3.80
3.65
2.51
2.44
1.00
1.00
2.03
2.91
3.02
3.08
9.5 9.0 8.5 8.0 7.5 7.0 6.5 6.0 5.5 5.0 4.5 4.0 3.5 3.0 2.5 2.0 1.5 1.0 0.5 0.0
f1 (ppm)
3500
3000
2500
2000
1500
1000
500
0

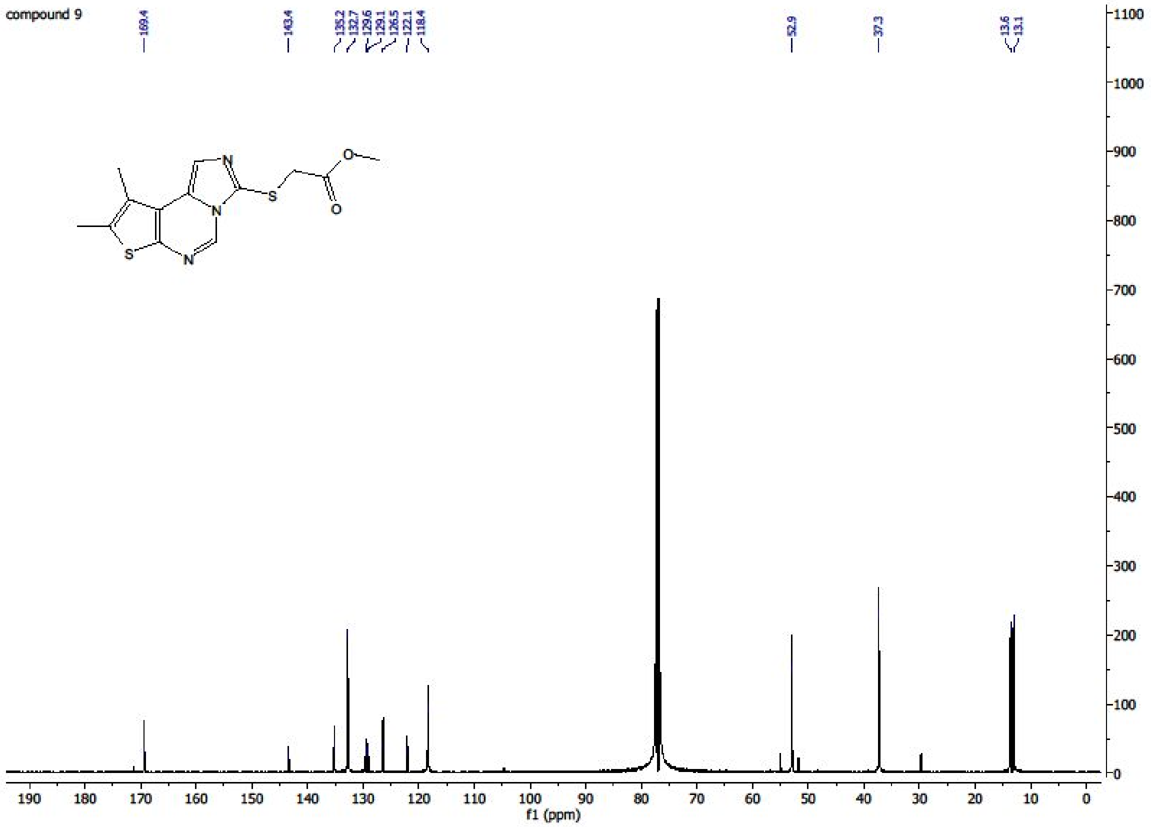
compound 9
169.4
143.4
135.2
132.7
129.6
129.1
126.5
122.1
118.4
52.9
37.3
13.6
13.1
190 180 170 160 150 140 130 120 110 100 90 80 70 60 50 40 30 20 10 0
f1 (ppm)
1100
1000
900
800
700
600
500
400
300
200
100
0

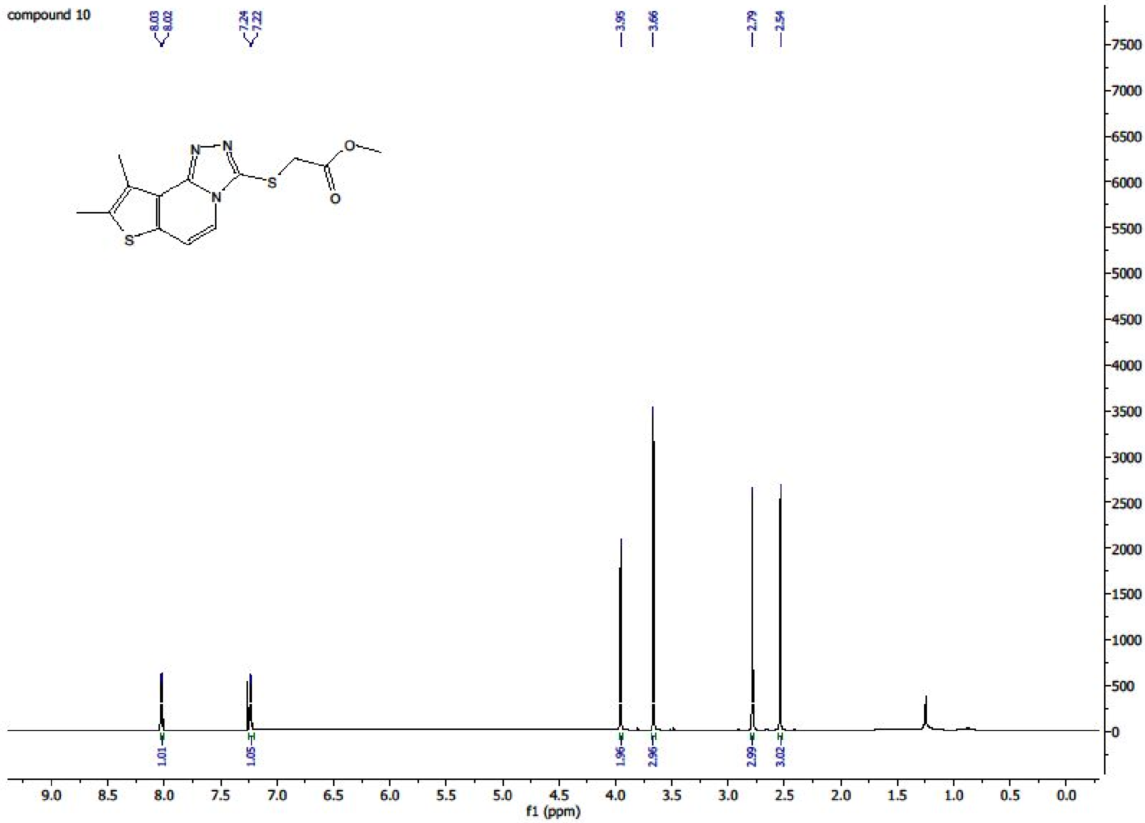
compound 10
8.03
8.02
7.24
7.22
3.95
3.66
2.79
2.54
1.01
1.05
1.96
2.96
2.99
3.02
f1 (ppm)

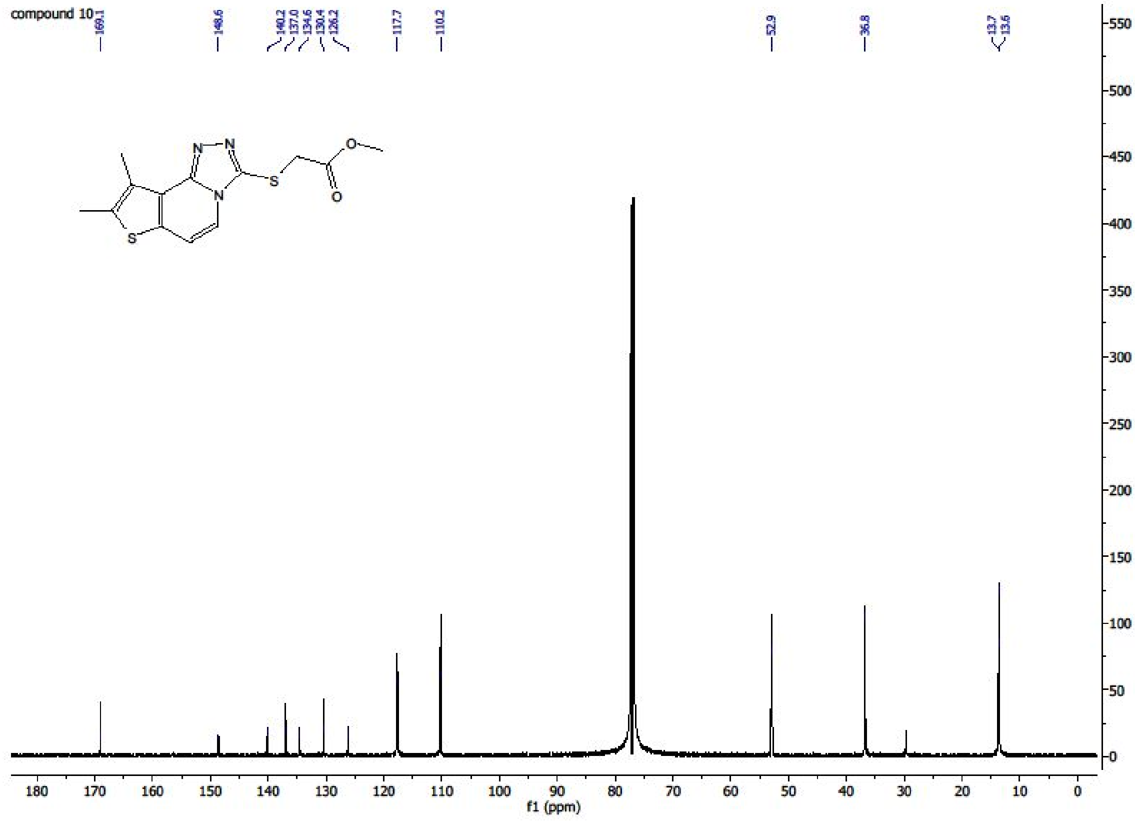
compound 10
169.1
148.6
140.2
137.0
134.6
130.4
126.2
117.7
110.2
52.9
36.8
13.7
13.6
f1 (ppm)
