# Supplementary material for: Cellular Target Deconvolution of Small Molecules Using a Selection-Based Genetic Screening Platform
Source: ACS Cent Sci. 2022 Sep 22;8(10):1424–34. doi: 10.1021/acscentsci.2c00609 (PMC9615120; doi:10.1021/acscentsci.2c00609)
Supplement: Supplementary file 4 — oc2c00609_si_004.pdf [file oc2c00609_si_004.pdf]

## Supporting information

# Cellular Target Deconvolution of Small Molecules using a Selection-based Genetic Screening Platform

Junxing Zhao[1], Zhichao Tang[1#], Manikandan Selvaraju[1#], Kristen A. Johnson[2], Justin T. Douglas[3], Philip F. Gao[4], H. Michael Petrassi[2], Michael Zhuo Wang[5], & Jingxin Wang[1]*

1 Department of Medicinal Chemistry, University of Kansas, Lawrence, KS 66047, USA
2 Calibr, Scripps Research Institute, La Jolla, CA 92037, USA
3 Nuclear Magnetic Resonance Laboratory, University of Kansas, Lawrence, KS 66047, USA
4 Protein Production Group, University of Kansas, Lawrence, KS 66047, USA
5 Department of Pharmaceutical Chemistry, University of Kansas, Lawrence, KS 66047, USA
*** Correspondence: wang.jingxin@ku.edu**

**Figure S1: BDW568 activation of IFN-I stimulated gene expression.**

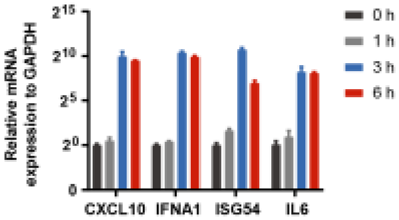


RT-qPCR analysis of type-I interferon stimulated genes' RNA expression level in THP-1 cells treated with BDW568. Representative result of two independent experiments with three biological replicates. Data were displayed as mean ± s.d.

**Figure S2: CRISPR screening conditions and results of known STING agonists 2’,3’-cGAMP and SR-717.**

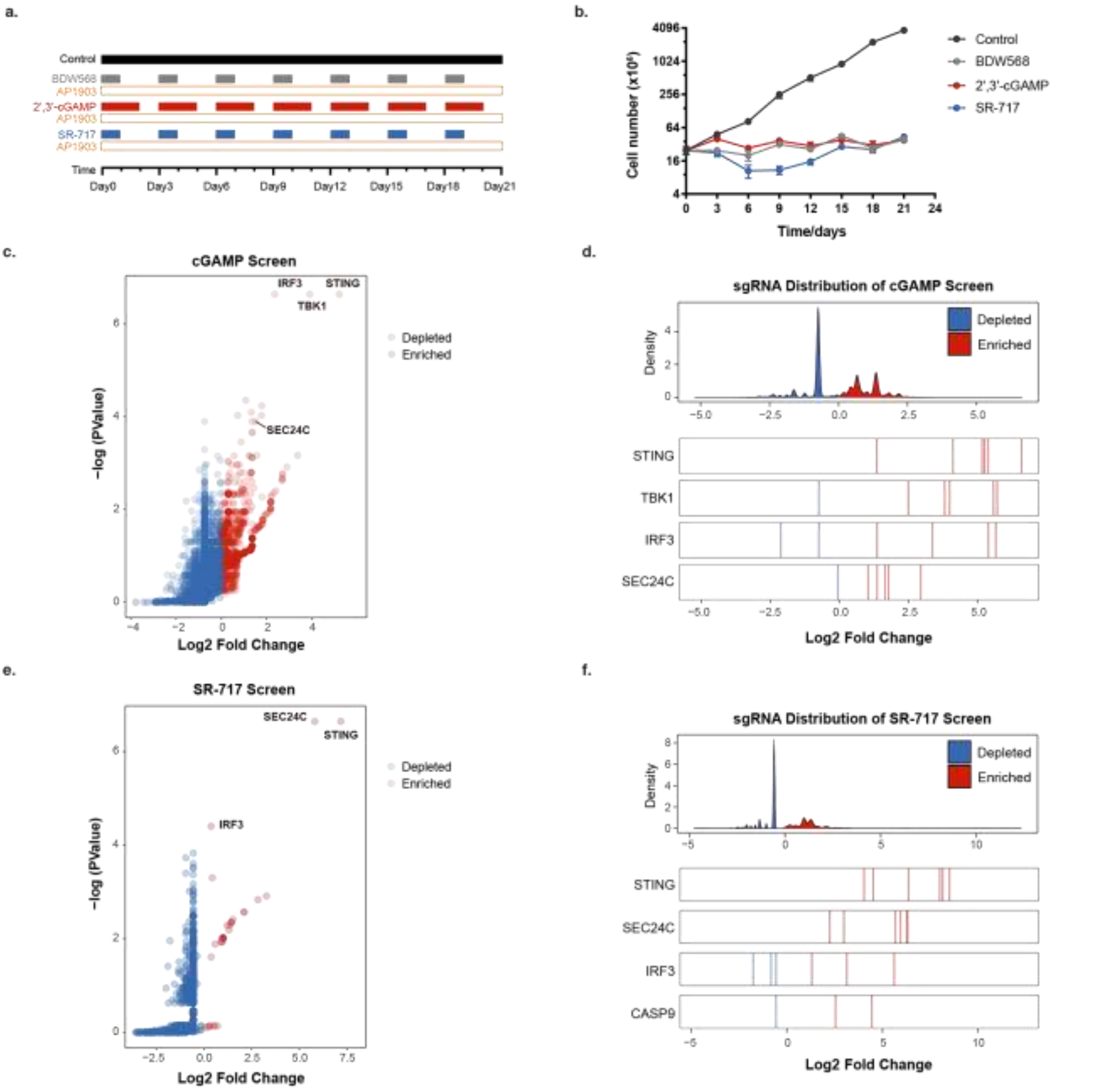


(a) The experimental schedule of CRISPR screen of BDW568, 2’,3’-cGAMP and SR-717. (b) Cell number was counted at the end of each cycle of BDW568, cGAMP or SR-717 combined with AP1903 selection. (c) & (e) Scatterplot for genes in 2’,3’-cGAMP CRISPR screening (c) and SR-717 CRISPR screening (e), (x-axis: median of log2(fold change) for all six sgRNAs per gene; y-axis: P-value calculated by MAGeCK’s positive algorithm; red dots: enriched genes; blue dots: depleted genes). (d) & (f) Top, frequency histogram of sgRNA fold change in 2’,3’-cGAMP group (d) or SR-717 group (f) comparing to non-treated cells for all sgRNAs. Bottom, distribution of log2(fold change) for the six sgRNAs targeting candidate genes identified in the 2’,3’-cGAMP CRISPR screening (d) and SR-717 CRISPR screening (f) (red lines: enriched; blue lines: depleted). Values are averaged over three biological replicates in (c), (d), (e), and (f).

## Figure S3: 2’,3’-cGAMP and SR-717 activity in THP-1 CES1 knockout cells

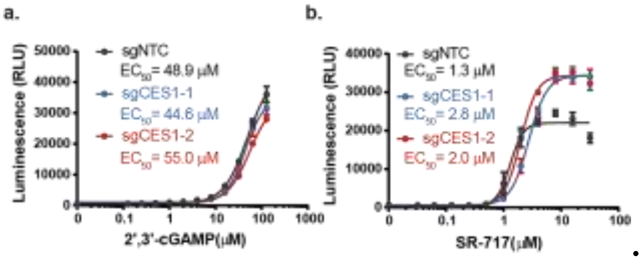


(a) ISRE-Luc activity of cGAMP was assessed in THP-1 cells with CES1 knockout by two separate sgRNAs, as well as non-targeting control sgRNA. (b) ISRE-Luc activity of SR-717 was assessed in THP-1 cells with CES1 knockout by two separate sgRNAs, as well as the non-targeting control sgRNA.

**Figure S4. Analysis of BDW568 metabolite in THP-1 cells.**

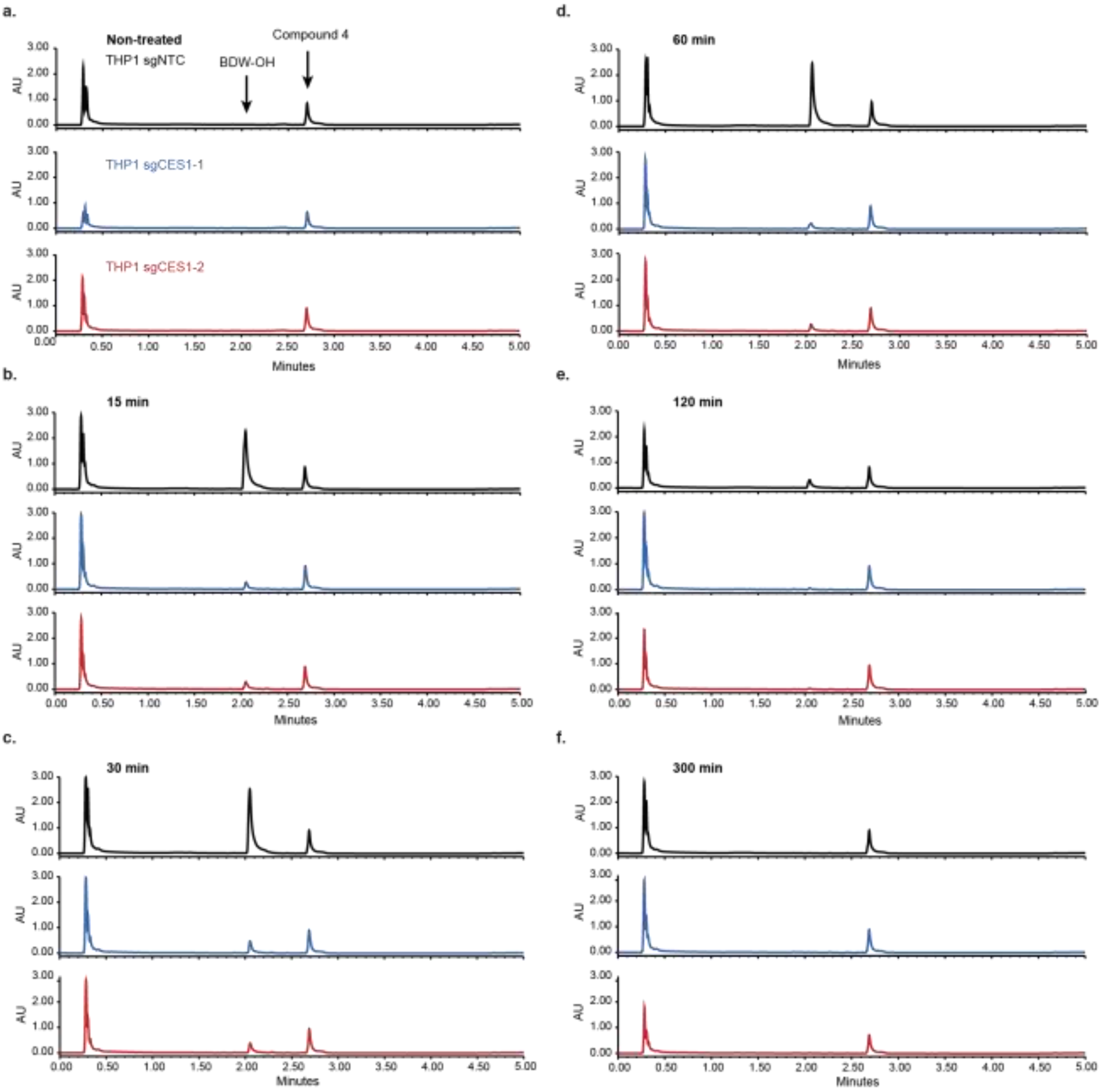


(a) Representative HPLC trace of non-treated samples (BDW568 was not added). (b)–(f) Representative HPLC trace of metabolite at 15–300 min after the addition of BDW568. Compound **4** was used as an internal standard.

**Figure S5: Purity of recombinant STING-AQ protein.**

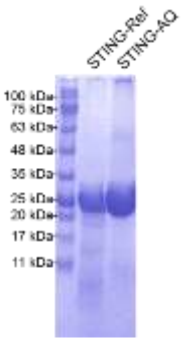


Coomassie staining of purified STING proteins after Tris-Glycine 16% SDS-PAGE. STING-Ref refers to STING (G230, R293) and STING-AQ refers to STING (A230, Q293). Data are representative of two independent experiments.

**Table S1: The activity of BDW568 and its analogues in ISRE THP-1 reporter assay.**

| Compound names | Structures | $EC_{50}$ (μM) |
|---|---|---|
| BDW568 | 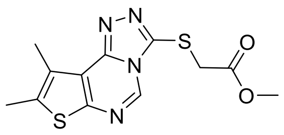 | 5.7 |
| BDW-OH (1) | 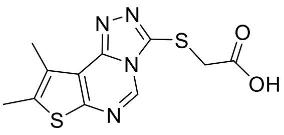 | inactive |
| BDW-NHMe (2) | 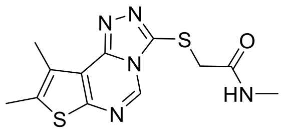 | inactive |
| 3 | 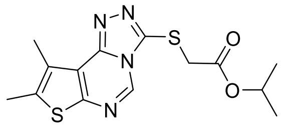 | 13.4 |
| BDW-O*t*Bu (4) | 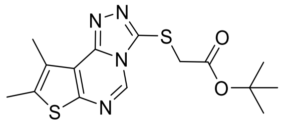 | inactive |
| 5 | 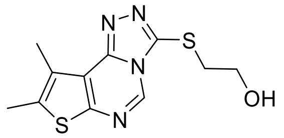 | inactive |
| 6 | 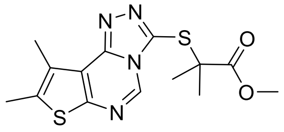 | inactive |
| 7 | 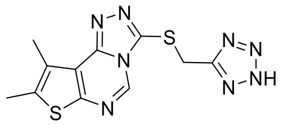 | inactive |
| 8 | 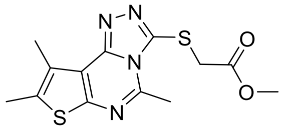 | inactive |
| 9 | 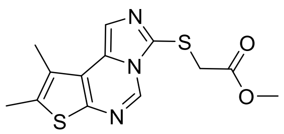 | inactive |
| 10 | 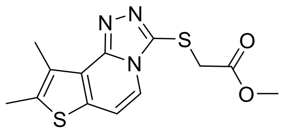 | inactive |

**Table S2: Enzyme kinetics study of recombinant CES1 on BDW568 hydrolyzation.**

| | CES1 | | | | PBS | | | |
|---|---|---|---|---|---|---|---|---|
| Conc. (μM) | Conversion rate | | | $v_{obs}$ (pM/min) | Conversion rate | | | $v_{obs}$ (pM/min) |
| 0.00 | 0.00 | 0.00 | 0.00 | 0.00 | 0.00 | 0.00 | 0.00 | 0.00 |
| 50.00 | 0.72 | 0.72 | 0.73 | 12.02 | 0.08 | 0.10 | 0.09 | 1.52 |
| 100.00 | 0.68 | 0.68 | 0.68 | 22.61 | 0.08 | 0.08 | 0.09 | 2.77 |
| 150.00 | 0.60 | 0.61 | 0.60 | 30.31 | 0.10 | 0.10 | 0.09 | 4.63 |
| 200.00 | 0.60 | 0.60 | 0.60 | 39.88 | 0.09 | 0.09 | 0.09 | 5.85 |
| 250.00 | 0.50 | 0.44 | 0.52 | 40.76 | 0.09 | 0.09 | 0.08 | 7.24 |
| 300.00 | 0.51 | 0.50 | 0.50 | 50.60 | 0.09 | 0.10 | 0.09 | 9.20 |
| 350.00 | 0.44 | 0.46 | 0.44 | 52.27 | 0.09 | 0.09 | 0.09 | 10.56 |
| 400.00 | 0.40 | 0.42 | 0.38 | 53.37 | 0.10 | 0.09 | 0.09 | 12.24 |
